# Are volatile methylsiloxanes in downcycled tire microplastics? Levels and human exposure estimation in synthetic turf football fields

**DOI:** 10.1007/s11356-024-31832-1

**Published:** 2024-01-16

**Authors:** Tiago Ferreira, Vera Homem, Francisco Cereceda-Balic, Ximena Fadic, Arminda Alves, Nuno Ratola

**Affiliations:** 1 LEPABE—Laboratory for Process Engineering, Biotechnology and Energy, Faculty of Engineering, University of Porto, Rua Dr. Roberto Frias, 4200-465 Porto, Portugal; 2 ALiCE—Associate Laboratory in Chemical Engineering, Faculty of Engineering, University of Porto, Rua Dr. Roberto Frias, 4200-465 Porto, Portugal; 3 Centre for Environmental Technologies (CETAM) and Department of Chemistry, Universidad Técnica Federico Santa María, Valparaíso, Chile

**Keywords:** End-of-life tires, Crumb rubber, Microplastics, Volatile methylsiloxanes, Tire additives, Human health

## Abstract

Downcycled rubber, derived from end-of-life tires (ELTs), is frequently applied as crumb rubber (CR) as infill of synthetic turf in sports facilities. This practice has been questioned in recent years as numerous studies have reported the presence of potentially hazardous chemicals in this material. CR particles fall into the category of microplastics (MPs), making them possible vectors for emerging micropollutants. A preliminary study where volatile methylsiloxanes (VMSs) were found in CR originated the hypothesis that VMSs are present in this material worldwide. Consequently, the present work evaluates for the first time the levels and trends of seven VMSs in CR from synthetic turf football fields, while attempting to identify the main sources and impacts of these chemicals. A total of 135 CR samples and 12 other of alternative materials were analyzed, employing an ultrasound-assisted dispersive solid-phase extraction followed by gas chromatography–mass spectrometry (GC–MS), and the presence of VMSs was confirmed in all samples, in total concentrations ranging from 1.60 to 5089 ng.g^−1^. The levels were higher in commercial CR (before field application), a reflection of the use of VMS-containing additives in tire production and/or the degradation of silicone polymers employed in vehicles. The VMSs generally decreased over time on the turf, as expected given their volatile nature and the wearing of the material. Finally, the human exposure doses to VMSs in CR (by dermal absorption and ingestion) for people in contact with synthetic turf in football fields were negligible (maximum total exposure of 20.5 ng.kg_BW_^−1^.year^−1^) in comparison with the European Chemicals Agency (ECHA) reference doses: 1.35 × 10^9^ ng.kg_BW_^−1^.year^−1^ for D4 and 1.83 × 10^9^ ng.kg_BW_^−1^.year^−1^ for D5. Nevertheless, more knowledge on exposure through inhalation and the combined effects of all substances is necessary to provide further corroboration. This work proved the presence of VMSs in CR from ELTs, another family of chemical of concern to take into account when studying MPs as vectors of other contaminants.

**Supplementary Information:**

The online version contains supplementary material available at 10.1007/s11356-024-31832-1.

## Introduction

It is estimated that approximately one billion end-of-life tires (ELTs) (i.e., rubber tires that can no longer be reused for its original purpose) are generated every year (WBCSD 2021). The handling of ELTs is a challenging task to waste management strategies due to their complex structure and composition (Xiao et al. 2022). Conventional disposal options, such as landfills and incineration, represent substantial environmental impacts. This eventually led the European Union (EU) to take legal action by banning the disposal of tires in landfills (*EC Directive 1999/31*; European Commission 1999) and establishing stricter incineration emission limits (*EC Directive 2000/76*; European Commission 2000). Consequently, sustainable management systems for ELTs were implemented, with remarkable results: about 91% of the ELTs generated in 2018 in 32 European countries were collected for recycling/downcycling and/or energy recovery (ETRMA 2020).

ELT downcycling consists of converting tires into a rubber granulate—a.k.a. crumb rubber (CR)—by grinding tire shreads and separating the rubber from the metal and textile components (Zafar 2021). This granulate can have numerous applications, including road paving, in the rubber and insulator industries, and, last but not least, in synthetic turfs of sports facilities (Valorpneu 2022), where it is used as infill to provide adequate cushioning and traction (HHS 2022).

The main components of a commercial tire are natural and synthetic rubber (43% w/w), reinforcing fillers (28% w/w), metals (particularly lead and cobalt) (13% w/w), aromatic oils (7.0% w/w), and textile material (5.0%) (ECHA 2017b), as well as dispersing agents and rubber vulcanization additives. The composition of ELTs is not expected to differ significantly from new tires, but some microcontaminants may be adsorbed or released during a tire’s lifetime (e.g., contact with the road surface) (Johannessen et al. 2022b). The most commonly reported hazardous chemicals in CR are polycyclic aromatic hydrocarbons (PAHs) (Armada et al. 2022; Celeiro et al. 2021a,b; De Groot et al. 2017; Halsband et al. 2020; Plesser and Lund 2004; Schneider et al. 2020a; Skoczyńska et al. 2021; Zhang et al. 2008), heavy metals (Marsili et al. 2015; Graça et al. 2022), and plasticizers (Armada et al. 2022; Celeiro et al. 2021a,b; De Groot et al. 2017; Halsband et al. 2020; Plesser and Lund 2004; Schneider et al. 2020a).

These compounds may present environmental and public health concern due to their harmful properties (Gomes et al. 2021). Nevertheless, only the Registration, Evaluation, Authorisation and Restriction of Chemicals (REACH)-8 PAHs are currently restricted in this matrix under international legislation (Zuccaro et al. 2022). An amendment issued by the EU in July, 2021 (and in effect since August, 2022), restricted the use of these PAHs in “granules or mulches” applied as “infill material in synthetic turf pitches” and/or “in loose form on playgrounds or in sport applications” to a maximum of 20 mg.kg^−1^ (*EC Regulation 1199/2021*; European Commission 2021). However, this limit is 20 times higher than for general consumer products, which raises some questions since playgrounds/sports field users come into frequent direct contact with CR.

Volatile methylsiloxanes (VMSs) are emerging organic pollutants that were included in the ECHA’s list of “substances of very high concern” (SVHC) in 2018 for being considered highly persistent and bioaccumulative (ECHA 2019). Moreover, there is evidence suggesting that some congeners could potentially act as endocrine disruptors (Lee et al. 2015), underlining the importance of its detection and quantification in materials in contact with humans and the environment. But to the authors’ best knowledge, they were never studied in CR. The presence of VMSs in tires could come both from the manufacturing process (incorporation of silicon-based additives) (Neethirajan et al. 2022) and later during the active period of the tires (degradation of silicone polymers applied in vehicles) (ECHA 2019). Indeed, industry reports mention the use of silicone materials (comprised of siloxane monomers) in numerous automobile parts, including tire surface modifiers (ShinEtsu 2017). As mentioned above, reinforcing fillers are one of the main components of tires, the most common being carbon black and silica (Shulman 2011; ECHA 2017b). The latter, unlike rubber, is a hydrophilic compound, and Brinke (2002) suggested that silane could be used as a coupling agent to promote adhesion between the two. The presence of silica-silane reinforcement in tires has now become a common practice (Neethirajan et al. 2022). When silanes bind to inorganic substrates, a series of hydrolysis and condensation reactions take place and a cross-linked siloxane structure is formed (Pape 2011). There are silane coupling agents containing methyl groups, which under certain conditions (e.g., high temperatures in industrial processes, contact with organic solvents) can contribute to the release of VMSs. For instance, cyclic VMSs are formed from the thermal degradation of silicones (Germain 2017). During the active lifetime of a tire, these chemicals could appear as impurities from the decomposition of silicone polymers employed in vehicle parts (e.g., body components, chassis) and in numerous automotive and tire care products (e.g., polishes, car wash detergents) (ECHA 2019). To the authors’ best knowledge, there is no obligation to report the use and quantities of VMSs in tire manufacturing additives, but their properties and extensive use suggest the application also in this material.

VMSs can also incorporate tire rubber during the mechanical grinding of tires to produce CR in rubber downcycling (e.g., from the contact with silicon-based lubricants applied in the granulators) and throughout CR’s lifetime. In this study, the application of this material in artificial turf football fields is addressed, and thus, the personal care products (PCPs) used by the players could be a relevant external source of VMSs. These chemicals (mainly the cyclic congeners D4, octamethylcyclotetrasiloxane; D5, decamethylcyclopentasiloxane; and D6, dodecamethylcyclohexasiloxane) are extensively employed in PCP formulations (Johnson et al. 2011), as well as in many other consumer and industrial products, such as silicone polymers, resins, and elastomers (Horii and Kannan 2020).

The European Chemicals Agency (ECHA) reported in 2017 that the human exposure to downcycled rubber granules was considered not prone to health risks (ECHA 2017a). Although these findings were later corroborated by other studies (De Groot et al. 2017; Peterson et al. 2018; Schneider et al. 2020b), ECHA stressed that this conclusion was not definitive, since there were still many uncertainties to be addressed, derived from the still limited number of studies for such a wide range of potential contaminants. The recent inclusion of CR in the group of microplastics (MPs) (ECHA 2021) has increased the concerns about this matrix. In fact, a more contemporary MP definition proposed by Verschoor (2015) includes tires in this class as they possess “synthetic material of environmental concern” (Lange et al. 2021; Verschoor et al. 2021). MPs are major transport vectors for environmental micropollutants (Gouin 2021), which means the potential accumulation of VMSs in CR must be taken into consideration, especially when in direct contact with humans, as is the case of synthetic turf football pitches.

All these pieces of evidence prompted our group to carry out preliminary screening tests on commercial samples of CR to verify the initial hypothesis of the presence of VMSs. The promising results obtained (positive qualitative confirmation of VMS chromatographic peaks) were the starting point of the present work. It addresses the current knowledge gaps in the composition of CR, a list of harmful constituents/additives that is constantly being updated in literature. The novelty of introducing a further class of microcontaminants that raises concern worldwide but was never studied in this material is a step forward toward the full comprehension of the potential negative impacts of CR to humans and the environment.

## Materials and methods

### Chemicals and reagents

The individual analytical standards of cyclic siloxanes (cVMSs) D3 (hexamethylcyclotrisiloxane), D4 (octamethylcyclotetrasiloxane), D5 (decamethylcyclopentasiloxane), and D6 (dodecamethylcyclohexasiloxane); and linear siloxanes (lVMSs) L3 (octamethyltrisiloxane), L4 (decamethytetrasiloxane), and L5 (dodecamethylpentasiloxane); the internal standard M4Q (tetrakis (trimethylsiloxy)-silane), and the QuEChERS (Quick, Easy, Cheap, Effective, Rugged, and Safe) sorbents C_18_ and primary-secondary amine (PSA) were acquired from Sigma-Aldrich (St. Louis, MO, USA). The solvents *n*-hexane (Hex), dichloromethane (DCM), and ethyl acetate (EtAc), with purities of 95.0%, 99.8%, and 99.7%, respectively, were purchased from VWR (Fontenay-sous-Bois, France), while magnesium sulfate (MgSO_4_) was from PanReac (Barcelona, Spain). Nitrogen (N_2_) for solvent blow-down and helium (He) as GC–MS carrier gas were supplied by Air Liquide (Maia, Portugal).

A detailed characterization of the seven target VMSs (including the nomenclature, chemical formula, molecular structure, and some relevant physicochemical properties) can be found in Supplementary Information (Table S1).

### Experimental procedure

#### Sampling

A total of 147 samples were analyzed. A total of 128 CR samples (120 outdoor and 8 indoor) and 12 samples of alternative infill material (cork, colored CR and a rubber/coconut synthetic fiber) were collected with a stainless steel gardening spade in synthetic turf fields from 15 different countries: Albania, Croatia, Estonia, Finland, France, Germany, Greece, Italy, Poland, Portugal, Serbia, Slovakia, Spain, Sweden, and Chile. Seven commercial (unused) CR samples supplied by manufacturers from China, Finland, and Portugal were included in the study to assess whether there is a change in the VMS content of CR derived from its use as turf infill. In most cases, the samples were conditioned in thermally stable cooling box until they were stored in the lab. Some samples were sent by post in similar conditions. In the lab, the samples were stored in paper envelopes and sealed in polypropylene (PP) bags on a special covert, at room (controlled) temperature and kept from sunlight, as in previous works with CR (e.g., Armada et al. 2022). Detailed information on the samples (identification code, country, type of pitch, pitch age, collection date, material, and granulometry) can be found in Supplementary Information (Table S2).

#### Method optimization

Being a pioneer study in targeting VMSs in CR, the validation of a reliable analytical method for their extraction and quantification from this matrix was required. The used approach was based on two existing protocols developed by our group: the first originally for the analysis of PAHs in CR (unpublished results), and the second to assess VMSs in solid sewage sludge (Silva et al. 2021). Some key parameters were tested (type of protocol, extraction solvent—from Hex, DCM and EtAc—solvent volume and type of syringe filter) through recovery assays with a VMS spike level of 500 ng.g^−1^. Duplicate laboratorial blanks were performed with every extraction batch. Finally, a modified approach of the first base protocol was selected. The diagram of the extraction procedure and the detailed results from the optimization assays can be found in Supplementary Information (section SI3).

#### Extraction procedure

First, 1.0 g of the CR samples and 500 ng of the internal standard M4Q were inserted in 15-mL silicon-free Falcon-type PP tubes. After 30 min (waiting time to allow the contact of the target analytes with the matrix), 5 mL of the extraction solvent, Hex/DCM 1:1 (v/v), was added. The mixture was vortexed for 1 min, sonicated for 20 min (J.P. Selecta bath, Barcelona, Spain), and centrifuged for 5 min at 4000 rpm (2670 g) (Rotofix 32A from Hettich, Kirchlengern, Germany). The supernatant was then moved to clean PP tubes, the QuEChERS sorbents (0.3 g MgSO_4_; 0.2 g PSA; 0.2 g C18) were added, and the mixture was vortexed for 1 min and centrifuged for 5 min at 2670 g. The liquid fraction was transferred to pre-baked 12-mL amber glass tubes and the volume reduced to about 2 mL under a gentle N_2_ flow. The extract was then filtered into GC vials with 0.2-µm pore size polytetrafluoroethylene (PTFE) syringe filters (VWR North America, Radnor, PA, USA) previously rinsed with Hex. The final volume was set to 1 mL under N_2_ prior to GC–MS analysis.

#### GC–MS analysis

The VMS quantification was performed in selected ion storage (SIS) mode with a Varian 4000 Ion Trap GC–MS (Palo Alto, CA, USA), in electron ionization (EI) mode (70 eV). A J&W DB-5 MS ultra-inert low-bleed capillary column (30 m length; 0.25 mm diameter; 0.25 µm film thickness) was used (Agilent, Santa Clara, CA, USA), and He was the carrier gas (1.0 mL.min^−1^). The oven temperature sequence was follows: 35 °C held for 5 min, then consecutive increases to 95 °C at 10 °C.min^−1^, to 140 °C at 5 °C.min^−1^, and to 300 °C at 35 °C.min^−1^ (held for 5 min)—total analysis time of 30 min. The sample injection (1 µL) was carried out in splitless mode in the first minute, then a split ratio of 100 from 1 to 5 min, and a split ratio of 5 from then on. The temperatures of the manifold (50 °C), injector (200 °C), ion trap (200 °C), and transfer line (250 °C) were constant and the filament was at 50 µA. The *m*/*z* ratios of the quantifier and qualifier ions and the retention times of each target chemical can be found in Supplementary Information (Table S3).

#### Method validation

Linearity, limits of detection (LOD) and quantification (LOQ), accuracy, and precision were the validation parameters performed. The VMS calibration curves comprised eight standards in Hex at concentrations ranging from 5 to 1000 µg.L^−1^ (containing the internal standard M4Q at 500 µg.L^−1^). The instrumental LODs and LOQs were calculated based on a signal-to-noise ratio (S/N) equal to 3 and 10, respectively. These limits were then used to calculate the method detection/quantification limits (MDLs/MQLs), taking into account the VMS recoveries. The accuracy was assessed with recovery assays of CR samples at two VMS spike levels: 100 and 500 ng.g^−1^ and the precision by the repeatability (or intra-day precision) and intermediate (or inter-day) precision at the same spike levels.

#### Quality assurance and quality control

The extensive use of VMSs in numerous consumer products (namely, PCP and GC–MS consumables) and the potential occurrence in the laboratory material required special safety measures to reduce/eliminate any external sample contamination. Non-volumetric glass material and the MgSO_4_ were baked at 450 °C overnight to eliminate volatile interferences. Before and after use, the microsyringes used to prepare the internal standard and the spike solutions were rinsed multiple times with acetone and Hex, and the needles for N_2_ blow-down were sonicated in acetone for 10 min. The silicon-free PP tubes used for the extractions were not reused. All non-essential PCPs, such as fragrances, hair gel, creams, soaps and lotions, were avoided by the lab analysts and silicone-free detergents were used for lab and material cleaning. The procedural blanks were performed in duplicate with every extraction batch and the results corrected accordingly whenever necessary. The mean blank levels were L3, not detected (n.d.); L4, below the method detection limit (< MDL); L5, n.d.; D3, < MDL; D4, 2.05 ± 1.13 ng.g^−1^; D5, 8.66 ± 3.31 ng.g^−1^; D6, 6.53 ± 2.21 ng.g^−1^. Instrumental blanks (direct injection of Hex) were run frequently during each GC–MS sequence to assess the potential VMS carryover in the column, which was not observed. A strict control of the behavior of M4Q (the most common internal standard used in the analysis of VMS when ^13^C-labeled congeners are not available) was done, since it was crucial for the quantification of the VMS levels. Highly constant recoveries around 60% were obtained in the extraction of samples, a very reasonable value when dealing with complex matrices. In the GC–MS, the injector was adapted with a Merlin microseal (instead of a conventional silicone rubber septum) and an ultra-inert column was used to minimize the bleeding of siloxanes. The method performance results are detailed in “Results and discussion” section.

#### Statistics

Statistical analysis was included in this study to infer the potential effect of several independent variables (country of origin, pitch age, type of field (i.e., indoor or outdoor), granulometry of the CR particles, and type of infill material) on the VMS content of the samples. A significance level of 95% was set for all tests, performed with the JASP open-source software (version 0.14.3, University of Amsterdam, The Netherlands; https://jasp-stats.org/).

To assess the normality of the data, the Shapiro–Wilk test was applied to the 128 field CR samples. A *p*-value of < 0.001 (below the significance threshold of 0.05) indicated a non-normal distribution. Consequently, non-parametric approaches were selected for the statistical analysis: Mann–Whitney (single variable; *N* = 2 subgroups) and Kruskal–Wallis tests (single variable; *N* > 2 subgroups) combined with Dunn post-hoc pairwise comparisons. Moreover, the Spearman correlation coefficient (*ρ*) was applied to assess potential correlations between the total concentrations of VMSs and of different families of microcontaminants in the CR samples in common (*N* = 53) with Armada et al. (2022) and Graça et al. (2022).

### Human exposure and risk

#### Model for human exposure estimation

The estimation of the human exposure to VMSs from CR in synthetic turf sports facilities was adapted from the methodology described by Peterson et al. (2018), which followed guidelines provided by the USA Environmental Protection Agency (US EPA) (EPA 1989). This study was selected since it also addresses hazardous chemicals (other than VMSs) present in synthetic turf CR. Some modifications were made in the exposure scenarios, pathways, and parameters to reflect the reality of football practice in Europe (from where most samples originate). Potentially confounding factors and the current lack of information on VMSs also imposed some limitations. In fact, the estimation of the inhalation of VMSs by the football players could carry a significant uncertainty, as the air composition may be largely affected by the PCPs employed by the sports facility users and/or by other nearby sources of VMSs. Thus, only the exposure through incidental CR ingestion and dermal absorption were considered in this study, as they are directly related to the VMS concentrations in the CR particles. Moreover, the exposure doses are only estimated for cVMSs, since there was no toxicological data available for lVMSs.

Three exposure scenarios/subjects were considered: young outdoor football players (6–11 years old), adult outdoor football players (> 18 years old), and pitch maintenance workers. For each scenario, both the expected exposure dose (the mean VMS concentration of all outdoor CR samples) and the “worst-case scenario” (the highest VMS concentration found in this study) were determined. The calculation of the oral and dermal VMS exposure doses (adapted from Peterson et al. 2018) was done with Eqs. S1 and S2, respectively (see Supplementary Information, section SI5). For the oral exposure, the relative bioaccessibility factors (*B*) for D4, D5, and D6 were obtained from ECHA’s database for toxicological properties (ECHA 2022a). Peterson et al. (2018) reported an ingestion rate (IR) of 50 mg.day^−1^ for incidental CR ingestion, which corresponds to half of the value in the US EPA’s *Directive for outdoor soil and dust ingestion* (EPA 2014). Being larger than dust, the probability of incidental ingestion of CR particles should be lower. To consider half of the value for soil and dust is debatable and may be an overestimation of the CR ingestion rate, but there was no other reference for CR studies. For the workers, the probability of CR ingestion was considered negligible in standard turf maintenance activities, so in this case only the exposure by dermal absorption was estimated. To simulate the exposure frequency (EF) under the average European conditions, it was assumed that in a year adult players use the fields 4 days a week in 3 h sessions during 11 months, young players 3 days a week in 3 h sessions during 10 months, and turf maintenance workers once a week in 8 h shifts during 11 months. This translates to approximately 24.0, 16.5, and 16.0 days.year^−1^, respectively. Finally, the mean body weight (BW) for 6–11 year olds and adults (31.8 and 80.0 kg, respectively) were taken from the US EPA’s *Exposure Factors Handbook* (EPA 2011).

The dermal absorption factors (ABS) for D4, D5, and D6 were also obtained from ECHA’s database (ECHA 2022a). A CR-to-skin adherence factor (AF) of 0.40 mg.cm^−2^—the mean value reported by the US EPA for football players (EPA 1989)—was adopted for the three groups. The exposed body surface area (SA) was calculated using the *Exposure factors handbook* (EPA 2011). This document contained, for different age groups, the mean total body surface area and the mean percent surface area of each body part. It was considered that the players’ body parts exposed were the head, forearms, hands, lower legs, and feet. According to the same reference, the forearms represented 45% of the arms and the lower legs 40% of the legs. All these assumptions carry some uncertainty. For instance, the clothes and other gear worn by the players may cover more or less the body (e.g., goalkeepers tend to have less skin exposed) and the body weight can be variable even within the same age group and gender.

#### Exposure risk

The risk associated with the VMS exposure in synthetic turf fields can be quantified by comparing the estimated exposure doses with the derived no-effect level (DNEL), a benchmark value above which humans should not be exposed (ECHA 2012). In this case, an exposure dose/DNEL ratio < 1 represents a safe exposure to humans. The DNEL values used in this study were taken from the ECHA toxicological database: D4, 1.35 × 10^9^ ng.kg^−1^.year^−1^; D5, 1.83 × 10^9^ ng.kg^−1^.year^−1^; D6, “no hazard identified” (ECHA 2022b). These values were found using a combined chronic toxicity/carcinogenicity study performed with rats, in which several physiological indicators were monitored for different exposure doses to create dose–response curves (OECD 2018). The experimental data was then extrapolated to humans by applying an overall assessment factor (OAF) which includes multiple parameters (e.g., duration of exposure, quality of the database, inter and intraspecies differences) (ECHA 2022b).

## Results and discussion

### Analytical method performance

The results obtained in the validation tests (detailed information in Supplementary Information—section SI6, Tables S4–S6) confirmed the effectiveness and reliability of the optimized analytical protocol. In fact, the MDLs and MQLs ranged from 0.006 (D5) to 4.46 ng.g^−1^ (D3) and from 0.018 (D5) to 14.9 ng.g^−1^ (D3), respectively. These low values allowed a very good detection of the target analytes in all samples. In terms of accuracy, the mean recovery from three replicates (for the seven target VMSs) was 104% for the 100 ng.g^−1^ spike and 98% for the 500 ng.g^−1^ spike, and within the 80–120% range commonly accepted in environmental analytics to disregard matrix effects. The precision parameters showed a repeatability (*N* = 5) and an intermediate precision (*N* = 3) with very low mean relative standard deviations (RSD) of 2.6% for 100 ng.g^−1^ and 3.9% for 500 ng.g^−1^, and 4.3% for 100 ng.g^−1^ and 2.5% for 500 ng.g^−1^, respectively. These performance indicators are similar or better than in other VMS studies in different matrices developed by our research group (e.g., Ramos et al. 2016; Rocha et al. 2019).

### Levels of VMSs in crumb rubber

The presence of VMSs was detected in all CR samples, with total concentrations ranging from 1.60 to 215 ng.g^−1^ in the synthetic turf field samples and from 90.2 to 5089 ng.g^−1^ in the commercial samples (Table 1). The cVMSs were always found at higher concentrations (max. 3912 ng.g^−1^ for D6) than the lVMSs, which were only detected in the commercial samples (max. 6.30 ng.g^−1^ for L5), as was the cyclic congener D3 (up to 216 ng.g^−1^). D5 was found in all samples, but also D6 had a very high frequency of detection (95.3% of samples), followed by D4 (75.8%). This is a common trend in literature for other matrices, which is expected given the estimated annual use in product formulations of D5 (16,266 tonnes), D6 (2,780 tonnes), and D4 (900 tonnes) (ECHA 2019).

**Table 1:** Percentage of detection, range and mean distribution profile (%), and concentrations of VMSs (ng.g^−1^) in crumb rubber collected from synthetic turf fields (F, *N* = 128) and commercial packages (C, *N* = 7)

Compound	% Detection		VMS distribution (%)			VMS concentration range (ng.g^−1^)	
	F	C	F	C		F	C
L3	0	85.7	0	n.d.–1.00 Mean: 0.31		n.d	n.d.–1.11 Mean: 0.53 ± 0.32
L4	0	14.3	0	n.d.–1.26 Mean: 0.18		n.d	n.d.–3.51 Mean: 3.51
L5	0	28.6	0	n.d.–1.94 Mean: 0.30		n.d	n.d.–6.30 Mean: 5.86 ± 0.25
D3	0	71.4	0	n.d.–14.7 Mean: 4.68		n.d	n.d.–216 Mean: 50.5 ± 75.6
D4	75.8	100	0–61.2 Mean: 7.86	2.09–50.8 Mean: 19.0		n.d.–23.0 Mean: 3.81 ± 2.64	10.4–142 Mean: 51.0 ± 51.6
D5	100	100	20.0–100 Mean: 59.0	16.7–75.9 Mean: 37.6		1.60–127 Mean: 23.4 ± 24.5	23.6–848 Mean: 191 ± 298
D6	95.3	100	0–62.4 Mean: 32.4	14.8–76.9 Mean: 37.9		n.d.–107 Mean: 13.9 ± 13.0	35.0–3912 Mean: 599 ± 1461
Total						1.60–215 Mean: 37.6 ± 15.0	90.2–5089 Mean: 880 ± 1858

*VMS*, volatile methylsiloxanes; *n.d.*, not detected

The detailed results from the VMS quantification of all 135 CR samples can be found in Supplementary Information (Table S7). As seen in Table 1, the VMS levels in commercial samples were considerably higher than in artificial turf CR, with mean concentrations of 880 ± 1858 and 37.6 ± 15.0 ng.g^−1^, respectively. This is a potential indicator that these chemicals are present in tire manufacturing, likely from the silanes added to bind the silica fillers to the rubber, or originate from the degradation/adsorption of silicone polymer products applied in automobiles. Sample C2 contributed significantly to these results, with a total VMS value of 5089 ng.g^−1^. This sample may have originated from tires that employed much more silicon-based products or additives in their manufacture or suffered from some contamination during the CR production process. But even without its contribution, the mean total VMS concentration was much higher (176 ± 88 ng.g^−1^; Table S7) than in turf samples. The lower concentrations found on field CR may be explained by the wear and tear of the material due to weather conditions and the activity of the pitch users, which may have led to some depletion of the VMSs by release to the atmosphere (more likely) or leaching, as explained above. This assessment is corroborated by samples CR206 (field of about 7 years, which may not correspond to the CR application time, depending on if and when the CR was replaced/refilled) and F1 (packed commercial sample before use in field 206): the total VMSs was almost three times higher in the unused CR (301 versus 106 ng.g^−1^).

The distribution profiles of individual VMS concentrations (Table 1) revealed similar trends within the field CR group, suggesting similar VMS sources. Overall, there was a predominance of D5, which has the highest mean presence in terms of congener profile (59%) and concentration (23.4 ± 24.5 ng.g^−1^) in the field samples, in line with the studies in other environmental matrices, often with high percentages—sludge from wastewater treatment plants (Bletsou et al. 2013), seawater (Horii et al. 2017), landfill leachates (Wang et al. 2020), and indoor air (Xiang et al. 2021). D6 followed in mean distribution value (32%) and total concentration (13.9 ± 13.0 ng.g^−1^), prevailing in some of the field CR samples, and D4 presence was the lowest, again in line with the production patterns. In general, the VMS profiles were more heterogeneous for the commercial CR than for the turf CR. In this case, D6 showed the highest mean (599 ± 1461 versus 191 ± 298 ng.g^−1^ for D5), although with very similar profile distribution (37.9 versus 37.6%, respectively). This result was again influenced by the very high D6 concentration (3912 ng.g^−1^) and fraction (76.9%) in sample C2, which likely incorporated (during tire or CR production) some D6-rich product. This trend of D6 predominance, although not so common, was found in literature, particularly in environmental matrices with a high organic content—raw sewage (interestingly in China, the origin of sample C2—Li et al. 2016; Zhang et al. 2020), pine needles, and soil (Ratola et al. 2016). D6 is the most lipophilic of the VMSs in study, which makes it prone to be linked with CR samples, and to have levels close to the prevailing D5.

Another important difference between the two sets of samples was the presence/absence of D3 and all the lVMSs (L3, L4, and L5), since these four compounds were only detected in commercial CR. Although at very low concentrations (in comparison to D4, D5, and D6), L3 was detected in six samples, L4 in one sample, L5 in two samples, and D3 in five samples. This can be indicative of VMS volatilization (predominantly for the smaller molecules D3 and L3) or even degradation processes occurring in this type of environment (Cheng et al. 2014; Grynkiewicz-Bylina et al. 2022). In fact, the more volatile and less lipophilic D3, L3, and D4 may be more subject to climate, either from exposure to the sun or leaching from rainfall, and periodical maintenance watering (especially in outdoor pitches), hence their lower (or non-detected) levels in field CR. This trend can be seen also for PAHs, where the lighter congeners were predominant in air sampled above the synthetic pitches (Dye et al. 2006; Schneider et al. 2020b) and in leachate from either field samples (Lim and Walker 2009; Celeiro et al. 2021b) and lab experiments (Plesser and Lund 2004; Cheng and Reinhard 2010).

These results reflect the difficulty in establishing very strong trends in this matrix. Commercial CR samples are produced in many different countries with processes and regulations that may differ significantly (Zuccaro et al. 2022). Moreover, the traceability of the ELTs used in such production is virtually impossible, as vehicles from a given country can use tires from worldwide brands. This also applies to the synthetic football pitches, where CR from a myriad of companies can be chosen, often depending only on the price. Semivolatile organic compounds (SVOCs) may suffer from external contaminations or release/transformation from products (e.g., Sukiene et al. 2016) and CR can be transported from very distant places until they reach the pitches. But this is also possible in CR from a nearby producer if a point source of the target chemicals has a direct impact. These uncertainties are very important and need to be taken into consideration, but are unfortunately almost impossible to quantify.

Comparing to the most representative families of other potentially hazardous compounds found in CR from synthetic turfs, the VMS levels were in the lower range. Literature reports mean concentrations overall from a minimum of 144 ng.g^−1^ for 7 PCBs (Plesser and Lund 2004) to a maximum of 76,300 ng.g^−1^ for the 16 EPA PAHs (De Groot et al. 2017), 89,570 ng.g^−1^ for 3 benzothiazoles (Schneider et al. 2020a), or 262,000 ng.g^−1^ for the 4 ECHA phthalates (Armada et al. 2022). Lately, novel compounds such as N-(1,3-dimethylbutyl)-N'-phenyl-*p*-phenylenediamine (6PPD), 1,3-diphenylguanidine (DPG), or hexamethoxymethylmelamine (HMMM)—additives used in the tire manufacturing industry—have been raising environmental concern (Johannessen et al. 2022a; Castan et al. 2023). So far, they were only targeted in CR in a single study (Schneider et al. 2020a), but yielding very high concentrations (570,530 ng.g^−1^ for 6PPD and 50,670 ng.g^−1^ for DPG). Only four CR samples in the present work (all commercial) had a total VMS concentration within this interval, with sample C2 clearly above the rest (5089 ng.g^−1^), followed by sample F1, with 301 ng.g^−1^. Recently, two studies evaluated the distribution of other micropollutants in CR in 53 coinciding samples with the present work (a summary of the results can be found in Supplementary Information, section SI8)—Armada et al. (2022) targeted the presence of 18 PAHs, 12 plasticizers (adipates, phthalates and bisphenol A), and 5 rubber additives (including benzothiazoles) and Graça et al. (2022) analyzed the levels of 30 metals and metalloids. The statistical significance of the relationships between the different classes of microcontaminants in both studies and VMSs (in the current work) in coinciding samples was checked using the Spearman correlation. The input data (i.e., the total concentrations of each class) and the Spearman *ρ* values (and *p*-values) obtained (one-to-one corresponding for the 53 coinciding samples in the three studies) can be found in Supplementary Information (Figures S5–S8 and Table S8, respectively). Results show that VMSs are significantly (and positively) correlated with additives (*ρ* = 0.479), possibly related to the incorporation of siloxanes and other silicone products in these additives, and PAHs (*ρ* = 0.381), which are found in numerous recycled rubber products (Diekmann et al. 2019). This suggests that VMSs may have similar sources in the tire manufacturing and that they may also be incorporated in CR during the tire lifetime, in line with the ability of CR to adsorb PAHs (Esfandiar et al. 2021). This potential uptake outside the manufacturing process is a very important parameter, not commonly addressed in literature (Trudsø et al. 2022). On the other hand, a negative significant correlation was found with plasticizers (*ρ* =  − 0.312). Since these additives and silicon polymers are applied in similar functions (e.g., to increase rubber flexibility), a higher use of one type could result in a lower need for the other, and vice versa. The same negative tendency is seen with metals, although not significant.

#### Distribution of VMSs by country

**Fig. 1:**
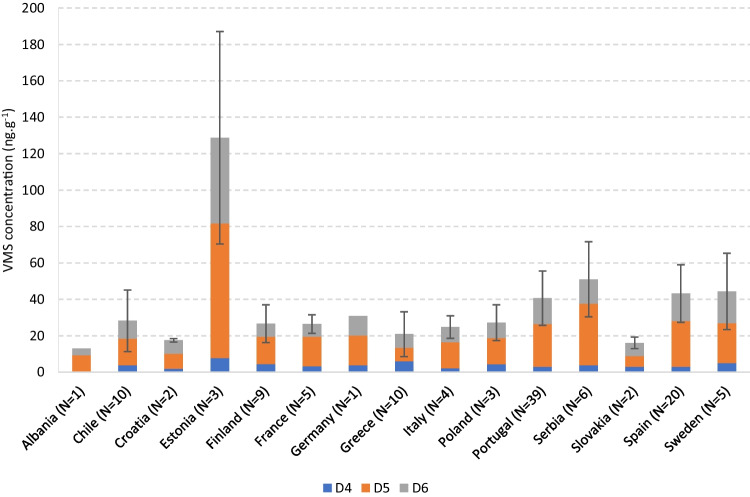
Mean concentration (ng.g^−1^) of the VMSs in crumb rubber from outdoor synthetic turf football fields, by country. N is the number of samples and the error bars represent the standard deviation within each subgroup and refer to the total VMS concentration

Since outdoor field CR samples were collected in 15 countries, it was assessed if geography was a determining factor in the presence of VMSs. Indoor samples were not considered here and are discussed in the next section. The mean total VMS concentration by country (with the respective standard deviation) is presented in Fig. 1.

The samples from Estonia clearly stood out, showing the highest mean VMS content (129.0 ± 58.4 ng.g^−1^), which could be explained by the peculiar weather conditions in this country. Before sample collection, it had been snowing for 2 weeks and the rubber granules were covered in snow for a long period of time. This likely caused a retention of the VMSs, as the snow present on the surface of the granules (along with the low air temperatures at the time) may have hindered both volatilization and leaching to the soil, as reported for other SVOCs (Herbert et al. 2006). Hence, these samples were considered separately in the statistical analysis henceforth. The countries that followed were Serbia (51.1 ± 20.6 ng.g^−1^), Sweden (44.6 ± 21.0 ng.g^−1^), Spain (43.2 ± 15.8 ng.g^−1^), and Portugal (40.1 ± 14.9 ng.g^−1^). On the other hand, the countries with the lowest total VMS levels were Albania (13.0 ng.g^−1^) and Slovakia (16.6 ± 3.14 ng.g^−1^) and the lowest individual concentration belonged to a sample from Greece (1.60 ng.g^−1^).

Since countries from different areas/climate conditions have similar mean levels, a trend was not evident. The statistical analysis confirmed some significant trends, but no clear impact of geography was inferred, given the contraditory findings. Initially, a Kruskal–Wallis test employed to the data of the countries with a minimum of two samples (*N* = 12) yielded a *p*-value of < 0.001, suggesting an effect of the pitch origin on the VMS composition. However, a Dunn post-hoc comparison evaluated the statistical proximity between countries (see Supplementary Information, Table S9), showing no immediately logical associations. In fact, countries separated by thousands of kilometers, or even continents (e.g., Chile and Italy (*p* = 0.912))—and with very distinct climate (e.g., Spain and Sweden (*p* = 0.882))—shared similar trends. But this also happened between neighbor countries: Portugal and Spain (*p* = 0.560), France and Italy (*p* = 0.878), or Poland and Slovakia (*p* = 0.380). On the other hand, significant differences were found between Croatia and Serbia (*p* = 0.017) or Sweden and Finland (*p* = 0.045), but also between Greece and Sweden (*p* = 0.009) or Finland and Spain (*p* = 0.003). In addition, as seen in Fig. 1, there were no significant differences in the profiles of the detected VMSs (D4, D5, and D6) in all countries, with D5 prevailing, followed by D6 and D4. This makes it difficult to differentiate the sources and is another indication that the origin of the pitches was not a relevant factor for the presence of VMSs.

Literature corroborates these findings. In total of 35 chemicals analyzed in CR, Armada et al. (2022) only reported the country of origin as a significant source of variation for 8 (4 PAHs, 3 plasticizers, and 1 additive) in 91 samples from 17 countries, and the results were insufficient to establish geography impacts on the levels of organic contaminants, given the CR traceability limitations. Graça et al. (2022) found the same evidence analyzing the levels of 30 metals and metalloids in 103 CR samples from 13 countries, suggesting that the pitch location was also not determinant for inorganic (metallic) species. For each country with the required number of samples (*N* ≥ 3; see Figs. S6–S8), the Spearman correlation did not reveal strong correlations nor common links, except in Finland between VMSs and PAHs and additives (Table S10), confirming the difficulties to relate the presence of contaminants in CR with the geography of the pitches.

As mentioned previously, the traceability of the CR used in a specific turf can be highly complex, mainly due to the fact that it is produced from downcycled tires that can originate from various (and predominantly untraced) countries. This may have a strong influence on the type, number, and level of contaminants in CR (Depaolini et al. 2017). The downcycling companies cannot assess the type (car, truck, or other) or manufacturer of the tires, and at what age they were ground into CR. For a more robust analysis, it would be important to have a representative selection of samples from all countries and improve the information on the origin of the CR, which was impossible to obtain for this work.

#### Indoor vs. outdoor facilities

**Fig. 2:**
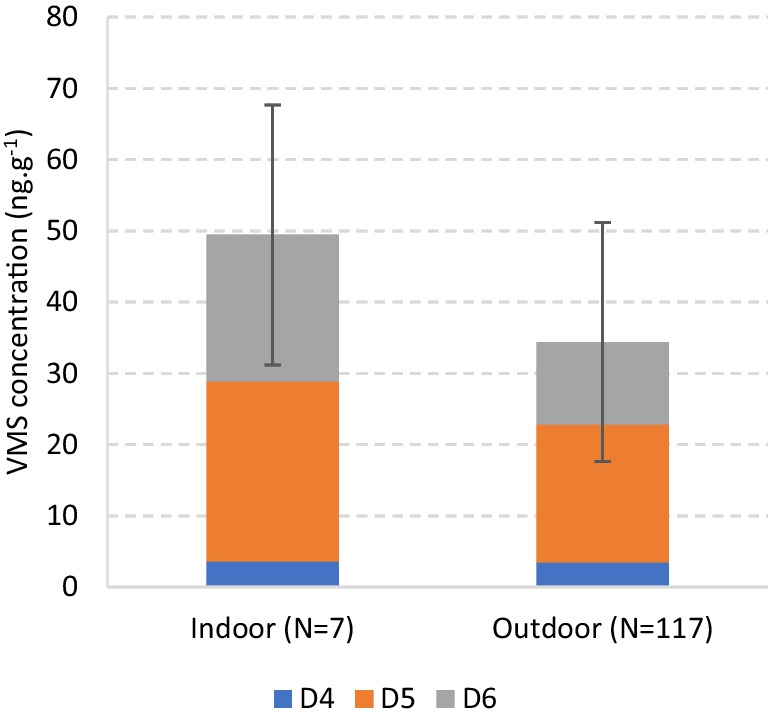
Mean concentration (ng.g^−1^) of VMSs in crumb rubber from indoor and outdoor synthetic football fields. N is the number of samples and the error bars represent the standard deviation within each subgroup and refer to the total VMS concentration

This study included 117 CR samples from outdoor and 7 from indoor pitches (without the samples from Estonia). Ideally, a larger number of indoor samples should be present. However, outdoor facilities are much more common, and therefore it was not possible to obtain a balanced set of samples. As seen in Fig. 2, the mean total VMS concentration was higher for the indoor (49.6 ± 18.3 ng.g^−1^) than for the outdoor samples (33.1 ± 16.8 ng.g^−1^).

Despite the much lower number of indoor samples, the Mann–Whitney test confirmed the statistical significance of this difference (*p* = 0.023). Nevertheless, a less uneven dataset should be used in future studies. The same trend was found in the samples from Estonia —the three outdoor samples showed lower VMS levels (128.9 ± 58.4 ng.g^−1^) than the single indoor sample (215 ng.g^−1^)—see Supplementary Information (Fig. S9). Although the literature is scarce on this subject, some studies have highlighted a similar trend between indoor and outdoor fields for different organic chemicals. In CR, Celeiro et al. (2021a, b) reported a mean total concentration of PAHs 3.1 times higher in indoor samples and Armada et al. (2022) found the same behaviour for pyrene (a PAH) and butyl benzyl phthalate (a plasticizer). In air sampled above the pitches, Ginsberg et al. (2011) and Simcox et al. (2011) also obtained higher concentrations of PAHs and VOCs in indoor facilities. On the other hand, heavy metals usually behave differently from organic compounds, and Graça et al. (2022) concluded that for the most relevant elements there were not statistical differences between the two types of fields.

According to the US EPA, the concentration of volatile organic compounds (VOCs) and SVOCs is expected to be higher indoors (EPA 2022), and the results agree with this assumption. This is essentially related to two factors: first, indoor fields are not directly exposed to sunlight, so the temperature of the pitch will be lower, and the volatilization rate of semi-volatile compounds like VMSs from CR slower; second, the commonly inadequate ventilation indoors will also limit the VMS release from the granules (mainly to the surrounding air).

Given the low number of indoor samples and the statistical differences observed between the two groups, only the outdoor CR samples were considered henceforth.

#### Granulometry

**Fig. 3:**
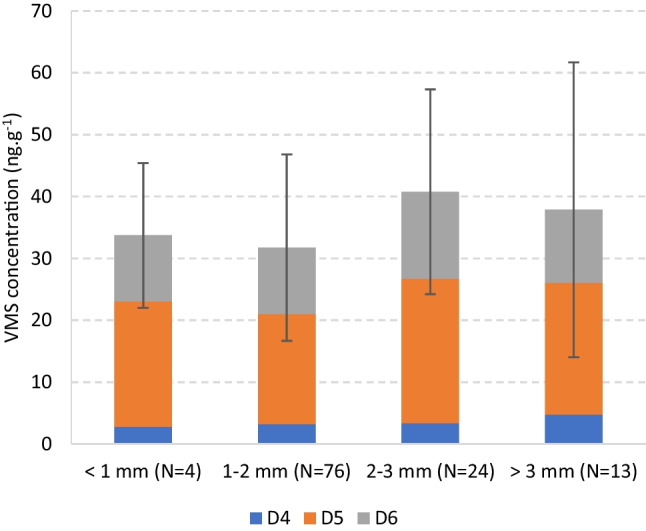
Mean concentration (ng.g^−1^) of VMSs in crumb rubber from outdoor synthetic turf football fields, by particle size. N is the number of samples and the error bars represent the standard deviation within each subgroup and refer to the total VMS concentration

The granulometry of the CR particles, calculated as the mean diameter of 20 random granules of each sample, ranged from 0.7 ± 0.2 (CR145) to 4.0 ± 1.2 mm (CR39). Except for a few granules in sample CR39, all others were smaller than 5 mm, matching the EU criteria for classification as microplastics (EU 2022) and the recent assessment of the scientific community (e.g., Luo et al. 2021). The data was divided into four subgroups: < 1 mm, 1–2 mm, 2–3 mm, and > 3 mm. Most samples were in the 1–2 mm group (*N* = 76), followed by the 2–3 mm group (*N* = 24). This reflects the mandatory size of CR granules for the infill of artificial turf pitches established by the International Federation of Association Football (FIFA)—from 0.8 to 2.5 mm (FIFA 2006). The mean total VMS concentrations by CR granule size are presented in Fig. 3, with values ranging from 30.1 ± 15.1 (1–2 mm) to 40.1 ± 16.6 ng.g^−1^ (2–3 mm). The individual VMS profiles were again similar between groups and with the same D5 > D6 > D4 trend.

A Kruskal–Wallis test performed on the four granulometry groups returned a *p*-value of 0.048 (< 0.05), indicating a slight but significant effect on the VMS concentration in CR. The Dunn post-hoc comparisons (see Supplementary Information, Table S11) indicated that the groups responsible for this difference were the 1–2 mm vs. 2–3 mm (*p* = 0.006). The latter had a higher VMS content, suggesting depletion as a result of the abrasion that may occur due to the wear and tear of the material from the continuous activity on the pitch (Gomes et al. 2021). This can also contribute to the reduction of the particle size in the same pitches with time. On the other hand, the surface area-to-volume ratio tends to be higher as the size decreases (Endo and Koelmans 2016), facilitating the sorption of organic contaminants (Bhagat et al. 2022). These counteracting factors and the variability of the sorption/desorption phenomena likely contribute for the similitude between size groups. In addition, the whole size range in this study may not be large enough to allow important changes in behavior, and the age of the CR is variable, which may have some effect. In other CR studies, Plesser and Lund (2004) and Graça et al. (2022) concluded that granulometry was not significant for the levels of PAHs and metals, respectively. The influence of granulometry is also not consensual for other microplastics. Fred-Ahmadu et al. (2020) reported a significant influence in the presence of other SVOCs, while some authors indicated that particle size effect was negligible (e.g., Chen et al. 2019 for dioxin-like chemicals).

#### Pitch age

**Fig. 4:**
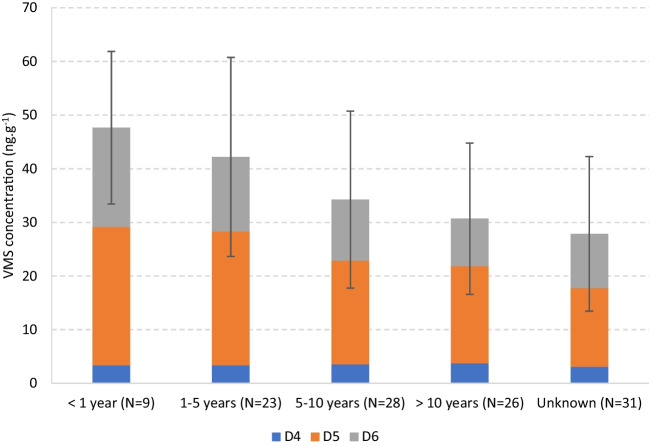
Mean total concentration (ng.g^−1^) and individual profiles of VMSs in crumb rubber from outdoor synthetic turf football fields, by pitch age. N is the number of samples and the error bars represent the standard deviation within each subgroup and refer to the total VMS concentration

Another variable evaluated was the age of the pitch, for which five subgroups were considered: < 1 year, 1–5 years, 5–10 years, > 10 years, and unknown (age not available). The results are presented in Fig. 4, with the mean total VMS concentration ranging from 26.1 ± 14.4 (unknown) to 45.8 ± 14.2 ng.g^−1^ (< 1 year).

As can be seen, the mean VMS levels decreased with the pitch age, more noticeably in the transition between the 1–5 year and 5–10 year subgroups. The samples from Estonia followed the same trend (see Supplementary Information, Fig. S11). The significance of the pitch age was confirmed by the Kruskal–Wallis test (*p* = 0.002) and the Dunn post-hoc comparisons (see Supplementary Information, Table S12) highlighted that the statistical proximity decreases with the age difference, which becomes a significant factor after 5 years (e.g., < 1 year vs. > 10 years (*p* = 0.014); 1–5 years vs. > 10 years (*p* = 0.026). The “unknown” subgroup was statistically different from all others except for the > 10 years, suggesting that most of its CR samples had been in the turf for several years. An influence of this variable was reported in literature for the presence of other microcontaminants in CR, mostly showing a decrease with the pitch age (e.g., Marsili et al. (2015) and Celeiro et al. (2021b) for PAHs; Donald et al. (2019) for methyl-PAHs; Zhang et al. (2008) and Ruffino et al. (2013) for some heavy metals). But Ruffino et al. (2013) and Marsili et al. (2015) also mentioned that this was not the case for some of the compounds they targeted. Given their volatile nature and the constant physical wearing of the material, it is expected that the concentration of VMSs in CR gradually decreases over time, although this decrease can be somewhat balanced by the potential adsorption of “new” VMS molecules, which may be present in the environment and/or the PCPs used by the players (Gomes et al. 2021). The individual VMS congener profiles showed no significant differences, but D4 and D5 increased their percentage with the pitch age, contrary to D6 (Fig. 4). D4 and D5 are more volatile, so they could be more available in the surrounding air to be absorbed by the CR granules. But overall, the results point toward a prevalence of VMS depletion over adsorption.

There is, however, a crucial factor that adds some uncertainty to this evaluation. Since in many facilities the infill CR is replaced/refilled occasionally, the age of the pitch may not correspond to the age of the sampled CR, nor to the time that the material has spent on it (Zhang et al. 2008). Consequently, despite the indication of possible impact of pitch age, the robustness of such assessments depends on keeping a strict record of the dates when the CR is replaced, which is not a common practice by field owners/workers.

### Concentration of VMSs in other materials

The present study included the analysis of 12 samples from 3 other materials applied as infill for artificial turf—colored/coated CR, cork, and a synthetic fiber of a rubber/coconut mixture. VMSs were detected in all samples, with total concentrations ranging from 7.79 (CR152, cork) to 99.2 ng.g^−1^ (CR126, colored CR)—see Table 2. The individual and total VMS concentrations of the analyzed samples are shown in Table S13 (Supplementary Information). D5 and D6 were present in all samples and, as in field CR, D3 and the lVMSs were not detected. As expected due to the different nature of the materials, a dispersion of the VMS levels between them was also found—mean totals of 55.4 ± 33.0 ng.g^−1^ for the colored CR, 27.1 ± 31.2 ng.g^−1^ for cork, and 73.7 ng.g^−1^ for the rubber/coconut fiber.

**Table 2:** Percentage of detection, and range and mean concentrations of VMSs in other materials (coloured/coated CR, cork and a synthetic fiber of a rubber/coconut mixture) collected from synthetic turf fields

Siloxane	% Detection	VMS concentration range (ng.g^−1^)			
		Colored rubber	Cork	Synthetic fiber	All alternative materials
L3	0	n.d	n.d	n.d	n.d
L4	0	n.d	n.d	n.d	n.d
L5	0	n.d	n.d	n.d	n.d
D3	0	n.d	n.d	n.d	n.d
D4	75	< MDL–3.73 Mean: 2.37 ± 1.27	< MDL–6.28 Mean: 2.14 ± 2.96	4.61	< MDL–6.28 Mean: 2.48 ± 1.93
D5	100	8.88–44.7 Mean: 26.1 ± 14.3	5.74–25.7 Mean: 13.3 ± 9.05	34.9	5.74–44.7 Mean: 22.6 ± 13.7
D6	100	1.95–50.9 Mean: 26.8 ± 17.8	1.17–41.6 Mean: 11.7 ± 19.9	34.2	1.17–50.9 Mean: 22.4 ± 18.6
Total		10.8–99.2 Mean: 55.4 ± 33.0	7.79–73.5 Mean: 27.1 ± 31.2	73.7	7.79–99.2 Mean: 47.5 ± 33.3

*VMS*, volatile methylsiloxanes; *n.d.*, not detected; *MDL*, method detection limit

To compare these alternative materials with the CR data (from field and commercial samples), a Kruskal–Wallis test was performed between the groups colored CR, cork, rubber/coconut fiber, field CR, and commercial CR (see Fig. S12, Supplementary Information). The results indicated a significant variation in the dataset (*p* < 0.001), but only between the commercial CR and the other groups, as seen by the Dunn post-hoc comparisons (*p* < 0.001 for field CR and cork, *p* = 0.033 for colored CR; see Supplementary Information, Table S14). These results were in line with the pitch age analysis, thus reinforcing that the VMS composition tends to decrease once the granules are placed in the synthetic pitches. Other studies reported significantly higher concentrations of hazardous chemicals in CR than in alternative infill (Armada et al. 2022; Celeiro et al. 2021b), but for VMSs the mean levels were slightly lower for CR (37.7 ± 28.7 ng.g^−1^) than for the other materials (47.5 ± 33.4 ng.g^−1^). The high value of the rubber/coconut fiber sample (comparing to the CR mean) is probably related to the process of blending both materials, in which silicon-based additives may have been used. Likewise, the colored CR could be indicative of a coating applied to the granules, also employing VMS-related additives. However, the use of coated CR apparently prevented the release of other organic chemicals to the environment, as reported Menichini et al. (2011) and Schneider et al. (2020a) for PAHs and some metals. But Gomes et al. (2010) concluded that different types of coating may have different responses.

Although a significant difference was not seen between cork and field CR (*p* = 0.201), likely due to the reduced number of cork samples, this natural matrix yielded the lowest VMS content overall. This evidence (expected being the only completely natural material analyzed) was also reported by Armada et al. (2022) and Celeiro et al. (2021b) for other organic microcontaminants, which supports the assumption that this material is a safer alternative to CR and potentially less hazardous to the users of synthetic turf facilities. Interestingly, this trend was not seen for heavy metals, except for Sb and As (Graça et al. 2022). Nevertheless, CR is still, by far, the first-choice material for infill, mainly due to the higher cost of cork (about four times per cubic meter) compared to CR (Gomes et al. 2021). It should be pointed out that the detection of VMSs in a natural material such as cork is a strong indicator that they were adsorbed during their lifetime on the pitch. This could be translated to CR, validating the assumption made in the previous sections that some adsorption of VMSs may take place after the application on the turf.

### Human exposure assessment

#### Exposure doses

From the results of the VMS quantification, it was possible to estimate human exposure doses to VMSs in outdoor CR by incidental ingestion and dermal absorption. Only D4, D5, and D6 were considered, as they were the ones detected in the field samples. The values for the different toxicological and exposure parameters can be found in Supplementary Information (Tables S15 and S16).

The estimated oral and dermal VMS exposure doses for adult players (AP), young players (YP), and turf maintenance workers (W) are presented in Table 3, both for the “expected” and “worst-case scenario” exposures. The oral exposure doses were, in all cases, higher than the dermal (up to 27 times). This indicates that the oral pathway through the incidental ingestion of CR, although much less frequent, may present a higher impact on the human exposure to VMSs from CR. There is a higher impact to young players than adults in the oral exposure (over 70% more), which is expected given the lower body weight and digestive metabolism processes. But almost no difference is found in the dermal exposure, since the surface of the body exposed to the VMSs and the time in contact with the turf is higher for adults. In the “worst-case scenario,” the exposure of adults is even prevails (0.80 vs. 0.72 ng.kg_BW_^−1^.year^−1^). The differences between the exposure doses in this extreme scenario and the “expected” conditions are quite high, particularly for the oral pathway (over 120 times). This means that a correct tracking and analysis of the CR to be used in sports facilities are recommended to avoid possible acute effects. The sports facility workers had the lowest dermal exposure due to the lower body surface exposed to the CR.

**Table 3:** Estimated doses (ng.kg_BW_^−1^.year^−1^) for oral and dermal exposure to VMSs from crumb rubber in outdoor synthetic turf football fields

Oral exposure								
Scenario	Expected				“Worst-case scenario”			
	D4	D5	D6	Total	D4	D5	D6	Total
AP	0.01	0.05	0.03	**0.09**	0.45	2.19	8.80	**11.4**
YP	0.02	0.09	0.05	**0.16**	0.77	3.79	15.22	**19.8**
W	Oral exposure not considered							
Dermal exposure								
Scenario	Expected				“Worst-case scenario”			
	D4	D5	D6	Total	D4	D5	D6	Total
AP	1.40 × 10^−3^	1.73 × 10^−2^	3.11 × 10^−5^	**0.02**	0.05	0.75	0.01	**0.80**
YP	1.27 × 10^−3^	1.56 × 10^−2^	2.81 × 10^−5^	**0.02**	0.04	0.67	0.01	**0.72**
W	9.35 × 10^−4^	1.16 × 10^−2^	2.07 × 10^−3^	**0.01**	0.03	0.50	0.01	**0.54**

*AP*, adult players; *YP*, young players; *W*, turf maintenance workers

As explained previously, it was not possible to account for the inhalation exposure, which could have an important impact, given the volatility of VMSs. In the absence of literature for VMSs in CR, a similar study by the ECHA reported that the inhalation exposure to PAHs (semi-volatile compounds) from CR was higher than the dermal but lower than the oral exposures (ECHA 2017a). The inhalation route may be predominant for more volatile chemicals, or change in importance depending on the location. In fact, Guo et al. (2021) mentioned that inhalation and dust ingestion were the main exposure pathways of children to VMSs in industrial areas, while in residential settings the dermal absorption prevailed. Still, the risk associated with CR may not be as high in comparison to some PCPs employing VMSs in their formulations at significantly higher levels (e.g., mean concentration of 151 µg.g^−1^ in facial creams and 4.21 µg.g^−1^ in deodorants; Capela et al. 2016).

#### Exposure risk

The experimental results from the combined chronic toxicity/carcinogenicity studies conducted by the ECHA show, for each exposure route, the systemic and local effects on the general population (ECHA 2022b). A long-term inhalation exposure to D4 and D5 caused respiratory tract irritation in the test subjects and a route-to-route extrapolation allowed the assessment of the oral exposure DNEL: 1.35 × 10^9^ ng.kg_BW_^−1^.year^−1^ for D4 (considering an OAF of 100) and 1.83 × 10^9^ ng.kg_BW_^−1^.year^−1^ for D5 (considering an OAF of 200). The OAF values were taken from the ECHA toxicological database, where little detail is given on the carcinogenicity studies conducted to estimate the DNELs for both chemicals. The document suggests that the OAF of D5 is higher due to a correction related to differences in the duration of exposure to both chemicals in the mentioned studies (ECHA 2022b). No hazards were identified from oral exposure to D6, nor via dermal exposure to any of the VMSs in question (D4, D5, and D6).

A comparison of the highest oral exposure dose mentioned in the previous section (“worst-case scenario” for young players) with the oral exposure DNEL (Table 4) clearly suggests that, despite their presence in CR, the VMS levels in this matrix are insufficient to cause any severe health repercussions from oral and dermal exposure to people who come in close contact with the synthetic turf pitches. However, these findings consider VMSs from an isolated impact perspective, and not considering their synergistic importance. Since the presence of many other classes of microcontaminants in CR is well documented, and there is already some evidence of potential hazardous effects of some chemicals in CR (e.g., Mann 2018; Pronk et al. 2020), it would be important to elaborate in the future a detailed study on the “cocktail effect” of their combined risk. This could potentially surpass the ECHA reference value, especially if the chemical interactions present an additive or exponential effect. But there is still a remarkable gap not only on the risks posed by CR (Watterson 2017) but also on the knowledge of the VMS exposure impacts, due to the scarce information available. More sampling campaigns in diverse environmental matrices, and more exposure and toxicological studies are needed to enhance the accuracy of these estimations. CR is used worldwide and a safe use for people in contact with it is needed to enforce the sustainability of ELT downcycling.

**Table 4:** Estimation of the exposure risk to VMSs from crumb rubber in outdoor synthetic turf football fields: highest reported oral exposure dose *vs.* ECHA’s general population DNEL (ng.kg_BW_^−1^.year^−1^)

“Worst-case scenario”			DNEL (general population)		
D4	D5	D6	D4	D5	D6
0.771	3.79	15.2	1.35 × 10^9^	1.83 × 10^9^	“No hazard identified”

## Conclusions

The present study was the first to target volatile methylsiloxanes (VMSs) in crumb rubber (CR)—an apparently sustainable material for the downcycling for millions of tonnes of previously discarded end-of-life tires (ELTs). Their presence was confirmed in a total of 135 CR samples (from synthetic football pitches and a few commercial ones) and 12 from alternative materials, in levels up to 5089 ng.g^−1^, adding VMSs to an already extensive list of microcontaminants found in this matrix. The mean total VMSs were 1.5 times higher in indoor pitches, and apparently there is a significant decrease in VMS levels with time after 5 years of the application in the pitches. This is mostly due to the wear and tear of the rubber particles. The country of the pitches and the granulometry of the CR particles had no significant impact. A human exposure assessment conducted at the likely the main exposure facilities to CR (synthetic turf football fields) showed that the combined oral and dermal risk associated with VMSs from CR is not relevant for football players and turf maintenance workers.

The hypothesis that VMSs could be constituents of CR since their properties and main uses were likely to include tire manufacturing additives was proven, and this was an important improvement on the state-of-the-art. To do so, a new analytical method based on an ultrasound-assisted dispersive solid-phase extraction with QuEChERS clean-up and GC–MS quantification was developed and validated, taking advantage of the group’s expertise with VMSs in other matrices (e.g., Ratola et al. 2016; Silva et al. 2021). The exposure risk assessment model adapted a methodology proposed by Peterson et al. (2018) to the European conditions of football practice and introduced the concept of risk not only to the players but also to the sports complex workers.

Nonetheless, this work presented some constraints. For instance, it was impossible to have an even number of samples between countries, type of CR (turf infill or commercial), and type of pitch (outdoor and indoor) that would add more robustness to the statistics analysis. Also, the source apportionment was not detailed without the possibility of tracking the tire rubber from the production until its grinding to CR, including the impact of the tire’s lifetime in a vehicle. Finally, the “true” exposure doses to VMSs in football fields are likely higher than the ones here presented, since toxicological databases currently provide limited data on VMSs and one of the main exposure routes for volatile compounds—inhalation—was not included due to lack of data. But even with these limitations, VMSs can now also be considered a part of CR, in response to the ECHA’s indication to address the current knowledge gap regarding the chemical composition of this matrix.

With CR now included in the family of microplastics, enough evidence is provided to enhance the awareness to the potential health and environmental impacts of this material, as many applications besides the turf infill are currently being adopted (e.g., playground mats, construction, and asphalt filler). In fact, there is already a call to an environmental regulation of tires to enforce sustainability hand in hand with circularity (Trudsø et al. 2022). Another option is the use of alternative (and safer) materials such as cork, which is still too expensive to be widespread. This would also reduce further harmful effects that arise from the release of a large amount of CR from synthetic turf pitches into the environment (Kole et al. 2023). It is thus important to continue the research on this area to fill the knowledge gaps and mitigate the uncertainty on the composition of CR and the potential toxicological properties of VMSs. For instance, the development and application of simpler methods for the determination in air (particularly indoors) may be of relevance to address the exposure by inhalation (important when volatile chemicals are involved). Another suggestion would be the development of a safe (and inexpensive) way to remove the harmful chemicals from CR within their production process.

### Supplementary Information

Below is the link to the electronic supplementary material.

Supplementary file1 (DOCX 3143 KB)

## Data Availability

The most relevant data generated or analyzed during this study are included in the main text and the Supplementary Information file. Further details may be available upon request.
