# Supplementary material for: Are volatile methylsiloxanes in downcycled tire microplastics? Levels and human exposure estimation in synthetic turf football fields
Source: Environ Sci Pollut Res Int. 2024 Jan 16;31(8):11950–67. doi: 10.1007/s11356-024-31832-1 (PMC10869416; doi:10.1007/s11356-024-31832-1)
Supplement: Supplementary file 1 — Supplementary file1 (DOCX 3143 KB) [file 11356_2024_31832_MOESM1_ESM.docx]

**SUPPLEMENTARY INFORMATION**

**Are volatile methylsiloxanes in downcycled tire microplastics? Levels and human exposure estimation in synthetic turf football fields**

**Tiago Ferreira^1,2^, Vera Homem^1,2^, Francisco-Cereceda-Balic^3^, Ximena Fadic^3^, Arminda Alves^1,2^, Nuno Ratola^1,2^***

^1^LEPABE – Laboratory for Process Engineering, Biotechnology and Energy, Faculty of Engineering, University of Porto, Rua Dr. Roberto Frias, 4200-465, Porto, Portugal

^2^ALiCE - Associate Laboratory in Chemical Engineering, Faculty of Engineering, University of Porto, Rua Dr. Roberto Frias, 4200-465 Porto, Portugal

^3^Centre for Environmental Technologies (CETAM) and Department of Chemistry, Universidad Técnica Federico Santa María, Valparaíso, Chile

*E-mail: nrneto@fe.up.pt

Number of pages: 28

Number of tables: 16

Number of figures: 12

**SI1 – Characterization of the analytes**

**Table S1 –** Nomenclature, chemical formula, molecular structure and physicochemical properties of the seven volatile methylsiloxanes (VMSs) analyzed in the present study.

| **VMS type** | **Full name**  **Abbreviated name**  **CAS No.** | **Formula** | **Structure** | **Molar mass (g.mol^-1^)** | **Boiling point (^o^ C)** | **Vapour pressure at 25 ^o^C (mmHg)** |
| --- | --- | --- | --- | --- | --- | --- |
| Linear VMS (lVMS) | Octamethyltrisiloxane  L3  107-51-7 | Si_3_—O_2_— (CH_3_)_8_ | 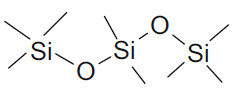 | 236.5 | 153.0 | 3.34 |
|  | Decamethytetrasiloxane  L4  141-62-8 | Si_4_—O_3_— (CH_3_)_10_ | 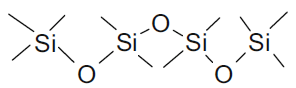 | 310.7 | 194.0 | 0.37 |
|  | Dodecamethylpentasiloxane  L5  141-63-9 | Si_5_—O_4_— (CH_3_)_12_ | 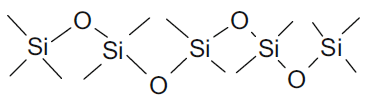 | 384.8 | 232.0 | 0.10 |
| Cyclic  VMS  (cVMS) | Hexamethylcyclotrisiloxane  D3  541-05-9 | Si_3_—O_3_— (CH_3_)_6_ | 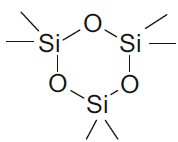 | 222.5 | 134.0 | 3.53 |
|  | Octamethylcyclotetrasiloxane  D4  556-67-2 | Si_4_—O_4_— (CH_3_)_8_ | 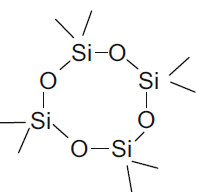 | 296.6 | 175.8 | 1.05 |
|  | Decamethylcyclopentasiloxane  D5  541-02-6 | Si_5_—O_5_— (CH_3_)_10_ | 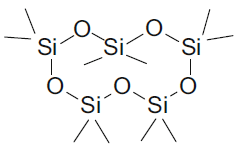 | 370.8 | 210.0 | 0.20 |
|  | Dodecamethylcyclohexa-siloxane  D6  540-97-6 | Si_6_—O_6_— (CH_3_)_12_ | 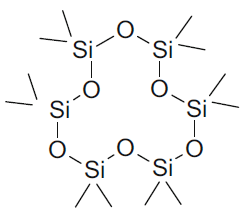 | 444.9 | 245.0 | 0.02 |

**SI2 – List of analysed samples**

**Table S2 –** List and details (ID, country of origin, type of pitch, pitch age, collection date, material and particle granulometry) of the 147 samples analyzed in the present study.

| **Sample ID** | **Country** | **Type of pitch** | **Pitch age** | **Collection date** | **Material** | **Granulometry (mm) ^(a)^** |
| --- | --- | --- | --- | --- | --- | --- |
| CR2 | Portugal | Outdoor | Unknown | 18/01/2017 | Crumb rubber | 1.36±0.20 |
| CR5 | Portugal | Outdoor | Unknown | 21/01/2017 | Crumb rubber | 1.78±0.40 |
| CR7 | Spain | Outdoor | Unknown | 22/01/2017 | Crumb rubber | 1.83±0.54 |
| CR15 | Portugal | Outdoor | Unknown | 07/02/2017 | Crumb rubber | 1.71±0.40 |
| CR21 | Portugal | Outdoor | 4 years | 12/02/2017 | Crumb rubber | 1.63±0.40 |
| CR23 | Portugal | Outdoor | 4 years | 14/02/2017 | Crumb rubber | 1.69±0.40 |
| CR25 | Portugal | Indoor | Unknown | 15/02/2017 | Crumb rubber | 1.71±0.36 |
| CR31 | Portugal | Outdoor | Unknown | 20/02/2017 | Crumb rubber | 1.96±0.43 |
| CR32 | Portugal | Outdoor | Unknown | 20/02/2017 | Crumb rubber | 2.97±0.81 |
| CR34 | Portugal | Outdoor | Unknown | 24/02/2017 | Crumb rubber | 1.00±0.20 |
| CR37 | Spain | Outdoor | Unknown | 06/03/2017 | Crumb rubber | 1.49±0.30 |
| CR39 | Spain | Outdoor | Unknown | 09/03/2017 | Crumb rubber | 3.96±1.18 |
| CR41 | Portugal | Outdoor | Unknown | 28/03/2017 | Crumb rubber | 2.09±0.41 |
| CR52 | Portugal | Outdoor | Unknown | 26/07/2017 | Crumb rubber | 3.44±0.76 |
| CR53 | Portugal | Outdoor | 7 years | 13/11/2017 | Crumb Rubber | 1.42±0.29 |
| CR55 | Portugal | Outdoor | 8 years | 17/11/2017 | Crumb Rubber | 1.22±0.29 |
| CR56 | Portugal | Outdoor | 8 years | 18/11/2017 | Crumb Rubber | 1.63±0.37 |
| CR59 | Portugal | Outdoor | Unknown | 17/12/2017 | Crumb Rubber | 1.50±0.34 |
| CR61 | Portugal | Indoor | 13 years | 23/12/2017 | Crumb Rubber | 1.64±0.25 |
| CR62 | Portugal | Outdoor | 1 year | 25/12/2017 | Crumb Rubber | 1.56±0.38 |
| CR63 | Portugal | Outdoor | Unknown | 25/12/2017 | Crumb Rubber | 1.14±0.27 |
| CR64 | Spain | Outdoor | Unknown | 28/12/2017 | Crumb Rubber | 1.27±0.27 |
| CR65 | Spain | Outdoor | 4 years | 28/12/2017 | Crumb Rubber | 1.76±0.30 |
| CR67 | Portugal | Outdoor | 14 years | 29/12/2017 | Crumb Rubber | 1.63±0.40 |
| CR69 | Portugal | Outdoor | 10 years | 29/12/2017 | Crumb Rubber | 1.46±0.29 |
| CR70 | Portugal | Outdoor | 1 year | 19/01/2018 | Crumb Rubber | 2.04±0.23 |
| CR71 | Portugal | Outdoor | 8 years | 19/01/2018 | Crumb Rubber | 1.25±0.30 |
| CR72 | Chile | Outdoor | 5 years | 11/04/2018 | Crumb Rubber | 1.48±0.39 |
| CR73 | Chile | Outdoor | Unknown | 12/04/2018 | Crumb Rubber | 1.60±0.23 |
| CR74 | Chile | Outdoor | Unknown | 12/04/2018 | Crumb Rubber | 1.96±0.36 |
| CR75 | Chile | Outdoor | Unknown | 12/04/2018 | Crumb Rubber | 2.00±0.39 |
| CR76 | Chile | Outdoor | Unknown | 12/04/2018 | Crumb Rubber | 1.92±0.31 |
| CR77 | Chile | Outdoor | 7 years | 16/04/2018 | Crumb Rubber | 0.97±0.24 |
| CR79 | Poland | Outdoor | Unknown | 30/08/2018 | Crumb Rubber | 1.99±0.29 |
| CR80 | Italy | Outdoor | 7 years | 12/09/2018 | Crumb Rubber | 1.48±0.31 |
| CR81 | Italy | Outdoor | 6/7 years | 17/09/2018 | Crumb Rubber | 1.78±0.29 |
| CR83 | Italy | Outdoor | 10 years | 18/09/2018 | Crumb Rubber | 1.53±0.37 |
| CR84 | Italy | Outdoor | 6/7 years | 18/09/2018 | Crumb Rubber | 1.90±0.30 |
| CR85 | France | Outdoor | 2-3 months | 29/09/2018 | Crumb Rubber | 2.19±0.45 |
| CR86 | France | Outdoor | 5 years | 30/09/2018 | Crumb Rubber | 1.88±0.37 |
| CR90 | Portugal | Outdoor | 20 years | 28/10/2018 | Crumb Rubber | 1.70±0.28 |
| CR92 | Portugal | Outdoor | 10 years | 02/11/2018 | Crumb Rubber | 1.75±0.35 |
| CR94 | Portugal | Outdoor | Installed but unused | 02/11/2018 | Crumb Rubber | 1.62±0.41 |
| CR97 | Portugal | Outdoor | 10 years | 03/11/2018 | Crumb Rubber | 1.68±0.42 |
| CR100 | Portugal | Outdoor | 2 years | 03/11/2018 | Crumb Rubber | 1.59±0.34 |
| CR101 | Spain | Outdoor | 14 years | 20/11/2018 | Crumb Rubber | 1.05±0.35 |
| CR102 | Spain | Outdoor | < 1 year | 20/11/2018 | Crumb Rubber | 1.14±0.33 |
| CR103 | Spain | Outdoor | 5 years | 20/11/2018 | Crumb Rubber | 1.18±0.29 |
| CR104 | Spain | Outdoor | 4 years | 20/11/2018 | Crumb Rubber | 1.68±0.42 |
| CR106 | Spain | Outdoor | 1 year | 23/11/2018 | Crumb Rubber | 1.42±0.34 |
| CR107 | Spain | Outdoor | 12-20 years | 23/11/2018 | Crumb Rubber | 1.51±0.36 |
| CR108 | Portugal | Outdoor | 6 years | 30/11/2018 | Crumb Rubber | 1.71±0.25 |
| CR110 | Portugal | Outdoor | 6 years | 09/12/2018 | Crumb Rubber | 1.54±0.40 |
| CR112 | Portugal | Outdoor | 11 years | 09/12/2018 | Crumb Rubber | 1.82±0.35 |
| CR114 | Portugal | Outdoor | Unknown | 30/12/2018 | Crumb Rubber | 1.74±0.40 |
| CR116 | Croatia | Outdoor | 9 years | 12/02/2019 | Crumb Rubber | 0.93±0.27 |
| CR117 | Croatia | Outdoor | 2 years | 15/02/2019 | Crumb Rubber | 1.44±0.40 |
| CR120 | Portugal | Outdoor | 2 years | 02/03/2019 | Crumb Rubber | 1.62±0.27 |
| CR121 | Portugal | Outdoor | 6 months | 02/03/2019 | Crumb Rubber | 1.59±0.35 |
| CR122 | Portugal | Outdoor | 10 years | 02/03/2019 | Crumb Rubber | 1.11±0.39 |
| CR123 | Portugal | Outdoor | 4/5 years | 02/03/2019 | Crumb Rubber | 1.67±0.26 |
| CR124 | Portugal | Indoor | 2 years | 02/03/2019 | Crumb Rubber | 1.59±0.31 |
| CR127 | Spain | Outdoor | 10 years | 26/04/2019 | Crumb Rubber | 1.81±0.40 |
| CR128 | Spain | Outdoor | 12 years | 27/04/2019 | Crumb Rubber | 1.81±0.37 |
| CR130 | Spain | Outdoor | Installed but unused | 03/05/2019 | Crumb Rubber | 1.61±0.32 |
| CR131 | Spain | Outdoor | 10 years | 03/05/2019 | Crumb Rubber | 1.68±0.42 |
| CR132 | France | Outdoor | 8 years | 12/05/2019 | Crumb Rubber | 1.09±0.22 |
| CR134 | France | Outdoor | 10 years | 17/05/2019 | Crumb Rubber | 1.63±0.36 |
| CR135 | France | Outdoor | 5 years | 17/05/2019 | Crumb Rubber | 2.37±0.54 |
| CR136 | Finland | Outdoor | 3 years | 27/05/2019 | Crumb Rubber | 2.16±0.91 |
| CR137 | Finland | Outdoor | 10 years | 28/05/2019 | Crumb Rubber | 2.08±1.28 |
| CR138 | Finland | Outdoor | Unknown | 28/05/2019 | Crumb Rubber | 1.74±0.41 |
| CR139 | Finland | Outdoor | 5 years | 28/05/2019 | Crumb Rubber | 2.85±0.60 |
| CR140 | Finland | Indoor | 5 years | 28/05/2019 | Crumb Rubber | 2.27±0.82 |
| CR141 | Finland | Outdoor | 5 years | 28/05/2019 | Crumb Rubber | 3.01±0.96 |
| CR142 | Finland | Outdoor | 5 years | 29/05/2019 | Crumb Rubber | 2.02±0.59 |
| CR144 | Finland | Outdoor | 2 years | 29/05/2019 | Crumb Rubber | 3.40±0.86 |
| CR145 | Finland | Outdoor | 10 years | 29/05/2019 | Crumb Rubber | 0.68±0.18 |
| CR146 | Finland | Outdoor | 1 year | 29/05/2019 | Crumb Rubber | 3.18±0.54 |
| CR148 | Spain | Outdoor | 9 years | 11/06/2019 | Crumb Rubber | 1.55±0.30 |
| CR154 | Greece | Outdoor | 10 years | 18/06/2019 | Crumb Rubber | 3.28±0.60 |
| CR155 | Greece | Outdoor | 10 years | 18/06/2019 | Crumb Rubber | 2.95±0.74 |
| CR156 | Greece | Outdoor | 10 years | 18/06/2019 | Crumb Rubber | 2.69±0.84 |
| CR159 | Portugal | Outdoor | Unknown | 26/06/2019 | Crumb Rubber | 1.36±0.30 |
| CR162 | Portugal | Outdoor | 15 years | 04/07/2019 | Crumb Rubber | 1.64±0.31 |
| CR164 | Spain | Outdoor | 20 years | 05/08/2019 | Crumb Rubber | 1.41±0.32 |
| CR166 | Spain | Outdoor | 5 years | 06/08/2019 | Crumb Rubber | 1.80±0.42 |
| CR167 | Spain | Outdoor | Installed but unused | 06/08/2019 | Crumb Rubber | 1.26±0.24 |
| CR168 | Portugal | Outdoor | Installed but unused | 31/08/2019 | Crumb Rubber | 1.40±0.27 |
| CR171 | Sweden | Outdoor | Unknown | 08/09/2019 | Crumb Rubber | 2.41±1.13 |
| CR172 | Sweden | Outdoor | 2 years | 11/09/2019 | Crumb Rubber | 1.77±0.71 |
| CR173 | Sweden | Outdoor | 5/6 years | 11/09/2019 | Crumb Rubber | 2.11±0.97 |
| CR174 | Sweden | Outdoor | 1 year | 11/09/2019 | Crumb Rubber | 2.40±0.97 |
| CR175 | Sweden | Outdoor | 14/15 years | 11/09/2019 | Crumb Rubber | 0.94±0.29 |
| CR177 | Portugal | Outdoor | 19 years | 22/10/2019 | Crumb Rubber | 1.07±0.27 |
| CR179 | Spain | Outdoor | 7 years | 23/10/2019 | Crumb Rubber | 1.55±0.30 |
| CR182 | Poland | Outdoor | 6 years | 05/12/2019 | Crumb Rubber | 1.93±0.54 |
| CR183 | Poland | Outdoor | 6 months | 05/12/2019 | Crumb Rubber | 1.71±0.27 |
| CR184 | Albania | Outdoor | 5 years | 03/01/2020 | Crumb Rubber | 2.18±0.56 |
| CR186_1 | Chile | Outdoor | 1 year | 04/03/2020 | Crumb Rubber | 1.82±0.52 |
| CR186_2 | Chile | Outdoor | 5 years | 04/03/2020 | Crumb Rubber | 1.63±0.44 |
| CR187 | Chile | Indoor | 6 years | 05/03/2020 | Crumb Rubber | 1.45±0.18 |
| CR188 | Chile | Outdoor | 2 years | 11/03/2020 | Crumb Rubber | 2.14±0.33 |
| CR190 | Chile | Outdoor | 6 years | 05/03/2020 | Crumb Rubber | 1.22±0.24 |
| CR192 | Germany | Outdoor | 12 years | 08/05/2020 | Crumb Rubber | 1.43±0.36 |
| CR194 | Portugal | Indoor | 12 years | 21/07/2020 | Crumb Rubber | 1.46±0.32 |
| CR195 | Portugal | Indoor | Unknown | 21/07/2020 | Crumb Rubber | 0.83±0.18 |
| CR163 | Portugal | Outdoor | Unused Infill | 04/07/2019 | Crumb Rubber | 1.53±0.35 |
| CR197 | Serbia | Outdoor | Unknown | 01/12/2021 | Crumb Rubber | 2.90±0.66 |
| CR198 | Serbia | Indoor | Unknown | 01/12/2021 | Crumb Rubber | 2.81±0.65 |
| CR199 | Serbia | Outdoor | 3-5 years | 01/12/2021 | Crumb Rubber | 3.07±0.79 |
| CR200 | Serbia | Outdoor | Unknown | 01/12/2021 | Crumb Rubber | 3.30±0.60 |
| CR201 | Serbia | Outdoor | Unknown | 01/12/2021 | Crumb Rubber | 4.13±0.87 |
| CR202 | Serbia | Outdoor | 2 years | 30/11/2021 | Crumb Rubber | 2.62±0.61 |
| CR203 | Portugal | Outdoor | 1 year | 15/12/2021 | Crumb Rubber | 2.57±0.49 |
| CR204 | Slovakia | Outdoor | < 3 years | 10/2021 | Crumb Rubber | 3.13±0.79 |
| CR205 | Slovakia | Outdoor | 6-8 years | 10/2021 | Crumb Rubber | 2.88±0.58 |
| CR206 | Estonia | Outdoor | 6-7 years | 14/02/2022 | Crumb Rubber | 2.71±0.71 |
| CR208 | Estonia | Outdoor | 10 years | 14/02/2022 | Crumb Rubber | 2.44±0.60 |
| CR209 | Estonia | Outdoor | 10 years | 14/02/2022 | Crumb Rubber | 3.10±0.54 |
| CR210 | Estonia | Indoor | 5 years | 14/02/2022 | Crumb Rubber | 2.18±0.31 |
| CR211 | Greece | Outdoor | Unknown | 17/06/2022 | Crumb Rubber | 1.17±0.33 |
| CR212 | Greece | Outdoor | Unknown | 17/06/2022 | Crumb Rubber | 3.19±0.77 |
| CR213 | Greece | Outdoor | Unknown | 17/06/2022 | Crumb Rubber | 3.18±0.66 |
| CR214 | Greece | Outdoor | 10 years | 17/06/2022 | Crumb Rubber | 2.66±1.13 |
| CR215 | Greece | Outdoor | 20 years | 17/06/2022 | Crumb Rubber | 3.26±0.72 |
| CR216 | Greece | Outdoor | 4 years | 17/06/2022 | Crumb Rubber | 2.95±0.56 |
| CR217 | Greece | Outdoor | Unknown | 17/06/2022 | Crumb Rubber | 1.41±0.47 |
| CL1 | Italy | Outdoor | 1 year | 18/09/2018 | Coloured CR | 1.18±0.45 |
| CK1 | France | Outdoor | Installed but unused | 04/10/2018 | Cork | 1.12±0.32 |
| RC1 | Spain | Outdoor | 3 years | 17/10/2018 | Rubber/Coconut fibre | 1.98±0.41 |
| CL2 | Spain | Outdoor | 18 years | 22/11/2018 | Coloured CR | 1.40±0.32 |
| CL3 | Portugal | Outdoor | 20 years | 03/03/2019 | Coloured CR | 1.45±0.50 |
| CL4 | Spain | Outdoor | < 1 year | 26/04/2019 | Coloured CR | 1.62±0.28 |
| CL5 | France | Outdoor | 2 years | 12/05/2019 | Coloured CR | 1.51±0.32 |
| CK2 | Spain | Outdoor | 1 year | 13/06/2019 | Cork | 1.54±0.26 |
| CL6 | Spain | Outdoor | 10 years | 06/08/2019 | Coloured CR | 1.85±0.28 |
| CL7 | Spain | Outdoor | 10 years | 23/10/2019 | Coloured CR | 1.97±0.43 |
| CK3 | Chile | Outdoor | 1 month | 05/03/2020 | Cork | 1.36±0.28 |
| CK4 | Chile | Outdoor | Unused Infill | 05/03/2020 | Cork | 1.51±0.32 |
|  |  |  |  |  |  |  |
|  |  |  |  |  |  |  |
|  |  |  |  |  |  |  |
|  |  |  |  |  |  |  |
| P1 | Portugal | Commercial samples – unused | | 2019 | Crumb Rubber | 2.02±0.72 |
| P2 | Portugal |  |  | 09/2018 | Crumb Rubber | 1.78±0.71 |
| C1 | China |  |  | 02/2017 | Crumb Rubber | 5.94±1.36 |
| C2 | China |  |  | 11/2021 | Crumb Rubber | 2.11±0.70 |
| C3 | China |  |  | 12/2021 | Crumb Rubber | 1.15±0.32 |
| C4 | China |  |  | 12/2021 | Crumb Rubber | - 1. ±0.60 |
| F1 | Finland |  |  | 02/2022 | Crumb Rubber | 1.98±0.62 |

^(a)^ Data from measurements performed on 20 randomly selected granules (mean ± standard deviation).

**SI3 – Analytical method optimization**

***SI3.1 – Extraction protocol***

***
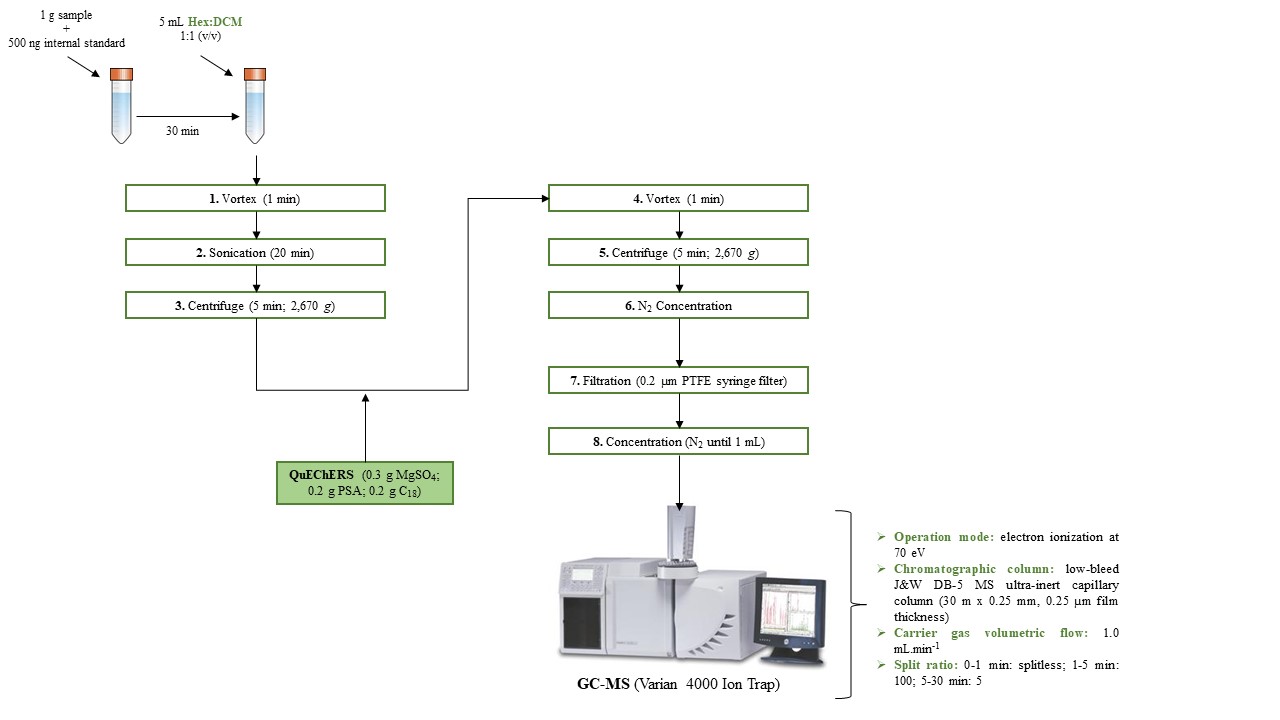
***

**Figure S1 –** Final protocol applied for the extraction of volatile methylsiloxanes from crumb rubber.

***SI3.2 – Recovery assays***

The first tests were to decide between two protocols previously developed at LEPABE: SG – intended for the analysis of PAHs in CR; DS1 – intended for the analysis of VMSs in dewatered sewage sludge.

Both protocols relied on an UAE with QuEChERS clean-up and extract filtration, followed by GC-MS instrumental analysis. The main differences between the two were the mass of sample (1.0 g in SG; 0.2 g in DS1), the duration of the sonication (20 min in SG; 10 min in DS1) and the mass of QuEChERS reagent C18 (200 mg in SG; 50 mg in DS1).

**Figure S2 –** Results from the recovery assays (n=4) carried out for the selection of the extraction solvent: n-hexane (Hex) vs. dichloromethane (DCM) vs. ethyl acetate (EtAc).

From this moment on, the DS1 protocol was chosen, and the remaining tests were done based on it.

**Figure S3 –** Results from the recovery assays (n=4) carried out for the selection of the extraction solvent (cont.): dichloromethane (DCM) vs. n-hexane/dichloromethane (Hex/DCM) 1:1 (v/v).

**Figure S4 –** Results from the recovery assays (n=4) carried out for the filtration step: 0.20 µm vs. 0.45 µm vs. no filtration (NF), using two solvent volumes (5 and 10 mL).

**SI4 – Instrumental analysis quantification parameters**

**Table S3 –** Retention times and quantifier/qualifier ions established for each of the VMSs analysed by GC-MS.

| **Compound** | **Retention time (min)** | **Quantifier ions (*m/z*)** | **Qualifier ions (*m/z*)** |
| --- | --- | --- | --- |
| D3 | 7.67 | 207 | 133, 191 |
| L3 | 8.89 | 221 | 73, 133 |
| D4 | 11.19 | 281 | 193, 265 |
| L4 | 12.66 | 207 | 73, 191, 295 |
| D5 | 14.45 | 267 | 73, 355 |
| M4Q (IS) | 15.19 | 281 | 73, 147, 369 |
| L5 | 16.57 | 281 | 73, 148, 369 |
| D6 | 18.49 | 341 | 73, 325, 429 |

**SI5 – Model for human exposure estimation**

**Equation S1 –** Estimation of human VMS intake levels by incidental crumb rubber ingestion – adapted from Peterson et al. (2018).

$$OED=\frac{C_{CR}\times B\times IR\times EF}{BW\times CF}$$

OED – oral exposure dose (mg.kg^-1^.year^-1^)

C_CR_ – concentration of VMSs in crumb rubber (mg.kg^-1^)

B – relative oral bioaccessibility factor (unitless)

IR – incidental crumb rubber ingestion rate (mg.day^-1^)

EF – exposure frequency (day.year^-1^)

CF – conversion factor (10^6^ mg.kg^-1^)

BW – average body weight of the target group (kg)

**Equation S2 –** Estimation of human VMS intake levels by dermal contact with crumb rubber – adapted from Peterson et al. (2018).

$$DED=\frac{C_{CR}\times ABS\times AF\times SA\times EF}{BW\times CF}$$

DED – dermal exposure dose (mg.kg^-1^.year^-1^)

ABS – dermal absorption factor (unitless)

AF – CR-to-skin adherence factor (mg.cm^-2^)

SA – exposed skin surface area (cm^2^.day^-1^)

**SI6 – Analytical method validation**

***SI6.1 – Limits of detection and quantification***

LOD and LOQ values were calculated based on a signal-to-noise ratio (S/N) equal to 3 and 10, respectively. These limits were determined for both the quantification instrument (IDL, IQL) and the analytical method (MDL, MQL).

**Table S4 –** Limits of detection and quantification for VMS analysis in crumb rubber.

| **Compound** | **IDL (pg)** | **IQL (pg)** | **MDL (ng.g^-1^)** | **MQL (ng.g^-1^)** |
| --- | --- | --- | --- | --- |
| L3 | 2.50 | 8.33 | 0.014 | 0.045 |
| L4 | 1.50 | 5.00 | 1.74 | 5.80 |
| L5 | 1.88 | 6.25 | 1.83 | 6.11 |
| D3 | 3.00 | 10.0 | 4.46 | 14.9 |
| D4 | 1.00 | 3.33 | 1.58 | 5.27 |
| D5 | 0.001 | 0.005 | 0.006 | 0.018 |
| D6 | 0.600 | 2.00 | 1.93 | 6.44 |

IDL – instrumental detection limit; IQL – instrumental quantification limit; MDL – method detection limit; MQL – method quantification limit.

***SI6.2 – Accuracy and precision***

**Table S5 –** Results of the accuracy tests (n=4) for VMS analysis in crumb rubber (at 100 ng.g^-1^ and 500 ng.g^-1^ spike levels).

| **Compound** | **Spike level (ng.g^-1^)** | **Rec_m_ (%)** |
| --- | --- | --- |
| L3 | 100 | 95 ± 2 |
|  | 500 | 98 ± 1 |
| L4 | 100 | 103 ± 6 |
|  | 500 | 100 ± 1 |
| L5 | 100 | 117 ± 7 |
|  | 500 | 100 ± 1 |
| D3 | 100 | 100 ± 3 |
|  | 500 | 107 ± 1 |
| D4 | 100 | 93 ± 2 |
|  | 500 | 92 ± 1 |
| D5 | 100 | 106 ± 9 |
|  | 500 | 92 ± 2 |
| D6 | 100 | 116 ± 8 |
|  | 500 | 95 ± 6 |

**Table S6 –** Results of the precision tests – repeatability (n=5) and intermediate precision (n=3) for VMS analysis in crumb rubber (at 100 ng.g^-1^ and 500 ng.g^-1^ spike levels).

| **Compound** | **Spike level (ng.g^-1^)** | **RSD - repeatability (%)** | **RSD – intermediate precision (%)** |
| --- | --- | --- | --- |
| L3 | 100 | 3.3 | 7.4 |
|  | 500 | 3.6 | 1.1 |
| L4 | 100 | 1.5 | 3.3 |
|  | 500 | 2.0 | 0.5 |
| L5 | 100 | 1.0 | 3.0 |
|  | 500 | 3.5 | 2.0 |
| D3 | 100 | 5.8 | 2.9 |
|  | 500 | 2.6 | 9.9 |
| D4 | 100 | 2.4 | 0.2 |
|  | 500 | 2.9 | 1.6 |
| D5 | 100 | 2.3 | 6.1 |
|  | 500 | 4.5 | 1.3 |
| D6 | 100 | 1.9 | 7.2 |
|  | 500 | 8.0 | 0.8 |

**SI7 – Concentration of VMSs in crumb rubber**

**Table S7 –** Concentration of VMSs in crumb rubber samples (ng.g^-1^). CR – samples from synthetic football pitches; P, C and F – commercial samples.

| **Sample ID** | **D3** | **D4** | **D5** | **D6** | **∑D3-D6** | **L3** | **L4** | **L5** | **∑L3-L5** | **Total** |
| --- | --- | --- | --- | --- | --- | --- | --- | --- | --- | --- |
| CR2 | n.d. | n.d. | 9.23 | 3.81 | 13.0 | n.d. | n.d. | n.d. | n.d. | 13.0 |
| CR5 | n.d. | n.d. | 11.5 | 2.75 | 14.2 | n.d. | n.d. | n.d. | n.d. | 14.2 |
| CR7 | n.d. | 3.25 | 2.22 | 4.39 | 11.3 | n.d. | n.d. | n.d. | n.d. | 11.3 |
| CR15 | n.d. | <MDL | 19.1 | 9.69 | 28.8 | n.d. | n.d. | n.d. | n.d. | 28.8 |
| CR21 | n.d. | n.d. | 15.6 | 4.04 | 19.6 | n.d. | n.d. | n.d. | n.d. | 19.6 |
| CR23 | n.d. | 3.19 | 16.0 | 10.2 | 29.7 | n.d. | n.d. | n.d. | n.d. | 29.7 |
| CR25 | n.d. | 6.67 | 29.8 | 17.7 | 54.4 | n.d. | n.d. | n.d. | n.d. | 54.4 |
| CR31 | n.d. | <MDL | 7.06 | 11.7 | 18.8 | n.d. | n.d. | n.d. | n.d. | 18.8 |
| CR32 | n.d. | 4.02 | 24.1 | 28.8 | 57.1 | n.d. | n.d. | n.d. | n.d. | 57.1 |
| CR34 | n.d. | <MDL | 11.2 | n.d. | 11.2 | n.d. | n.d. | n.d. | n.d. | 11.2 |
| CR37 | n.d. | n.d. | 9.97 | 3.44 | 13.4 | n.d. | n.d. | n.d. | n.d. | 13.4 |
| CR39 | n.d. | 1.91 | 10.2 | 5.97 | 18.2 | n.d. | n.d. | n.d. | n.d. | 18.2 |
| CR41 | n.d. | 1.81 | 8.00 | 7.34 | 17.4 | n.d. | n.d. | n.d. | n.d. | 17.4 |
| CR52 | n.d. | n.d. | 8.77 | 7.26 | 16.0 | n.d. | n.d. | n.d. | n.d. | 16.0 |
| CR53 | n.d. | <MDL | 12.2 | 3.38 | 15.6 | n.d. | n.d. | n.d. | n.d. | 15.6 |
| CR55 | n.d. | <MDL | 13.3 | 4.40 | 17.7 | n.d. | n.d. | n.d. | n.d. | 17.7 |
| CR56 | n.d. | <MDL | 12.0 | 2.89 | 14.9 | n.d. | n.d. | n.d. | n.d. | 14.9 |
| CR59 | n.d. | <MDL | 15.4 | 7.78 | 23.2 | n.d. | n.d. | n.d. | n.d. | 23.2 |
| CR61 | n.d. | 1.73 | 18.3 | 13.4 | 33.5 | n.d. | n.d. | n.d. | n.d. | 33.5 |
| CR62 | n.d. | 2.07 | 19.4 | 14.8 | 36.4 | n.d. | n.d. | n.d. | n.d. | 36.4 |
| CR63 | n.d. | 6.99 | 21.4 | 14.5 | 43.2 | n.d. | n.d. | n.d. | n.d. | 43.2 |
| CR64 | n.d. | <MDL | 17.5 | 5.00 | 22.5 | n.d. | n.d. | n.d. | n.d. | 22.5 |
| CR65 | n.d. | <MDL | 13.8 | 8.97 | 22.8 | n.d. | n.d. | n.d. | n.d. | 22.8 |
| CR67 | n.d. | <MDL | 9.43 | 2.32 | 11.8 | n.d. | n.d. | n.d. | n.d. | 11.8 |
| CR69 | n.d. | <MDL | 15.4 | 3.19 | 18.6 | n.d. | n.d. | n.d. | n.d. | 18.6 |
| CR70 | n.d. | <MDL | 18.4 | 6.20 | 24.6 | n.d. | n.d. | n.d. | n.d. | 24.6 |
| CR71 | n.d. | <MDL | 17.1 | <MDL | 17.1 | n.d. | n.d. | n.d. | n.d. | 17.1 |
| CR72 | n.d. | <MDL | 15.2 | 7.09 | 22.3 | n.d. | n.d. | n.d. | n.d. | 22.3 |
| CR73 | n.d. | 3.28 | 15.0 | 11.3 | 29.8 | n.d. | n.d. | n.d. | n.d. | 29.8 |
| CR74 | n.d. | 3.86 | 16.4 | 10.6 | 31.1 | n.d. | n.d. | n.d. | n.d. | 31.1 |
| CR75 | n.d. | 2.25 | 16.1 | 16.6 | 35.1 | n.d. | n.d. | n.d. | n.d. | 35.1 |
| CR76 | n.d. | <MDL | 12.6 | 10.3 | 22.9 | n.d. | n.d. | n.d. | n.d. | 22.9 |
| CR77 | n.d. | <MDL | 13.8 | 8.56 | 22.4 | n.d. | n.d. | n.d. | n.d. | 22.4 |
| CR79 | n.d. | <MDL | 19.0 | 12.0 | 31.0 | n.d. | n.d. | n.d. | n.d. | 31.0 |
| CR80 | n.d. | 2.11 | 11.8 | 5.80 | 19.8 | n.d. | n.d. | n.d. | n.d. | 19.8 |
| CR81 | n.d. | <MDL | 11.9 | 5.72 | 17.7 | n.d. | n.d. | n.d. | n.d. | 17.7 |
| CR83 | n.d. | 2.03 | 15.2 | 9.35 | 26.7 | n.d. | n.d. | n.d. | n.d. | 26.7 |
| CR84 | n.d. | 6.16 | 9.7 | 4.62 | 21.1 | n.d. | n.d. | n.d. | n.d. | 21.1 |
| CR85 | n.d. | 5.20 | 20.3 | 13.1 | 38.9 | n.d. | n.d. | n.d. | n.d. | 38.9 |
| CR86 | n.d. | 1.68 | 13.4 | 7.40 | 22.6 | n.d. | n.d. | n.d. | n.d. | 22.6 |
| CR90 | n.d. | 1.61 | 11.5 | 11.4 | 24.7 | n.d. | n.d. | n.d. | n.d. | 24.7 |
| CR92 | n.d. | <MDL | 9.00 | 5.97 | 15.0 | n.d. | n.d. | n.d. | n.d. | 15.0 |
| CR94 | n.d. | 2.79 | 23.9 | 25.3 | 52.1 | n.d. | n.d. | n.d. | n.d. | 52.1 |
| CR97 | n.d. | 4.56 | 16.9 | 10.8 | 32.5 | n.d. | n.d. | n.d. | n.d. | 32.5 |
| CR100 | n.d. | 5.62 | 25.7 | 8.68 | 40.2 | n.d. | n.d. | n.d. | n.d. | 40.2 |
| CR101 | n.d. | 4.00 | 6.14 | <MDL | 10.8 | n.d. | n.d. | n.d. | n.d. | 10.8 |
| CR102 | n.d. | 5.76 | 15.8 | <MDL | 21.9 | n.d. | n.d. | n.d. | n.d. | 21.9 |
| CR103 | n.d. | 3.70 | 11.3 | 9.34 | 24.7 | n.d. | n.d. | n.d. | n.d. | 24.7 |
| CR104 | n.d. | <MDL | 14.9 | 9.03 | 23.9 | n.d. | n.d. | n.d. | n.d. | 23.9 |
| CR106 | n.d. | 3.02 | 22.5 | 16.4 | 42.1 | n.d. | n.d. | n.d. | n.d. | 42.1 |
| CR107 | n.d. | 1.89 | 11.1 | 6.83 | 20.0 | n.d. | n.d. | n.d. | n.d. | 20.0 |
| CR108 | n.d. | 4.00 | 20.9 | 17.4 | 42.5 | n.d. | n.d. | n.d. | n.d. | 42.5 |
| CR110 | n.d. | 2.08 | 14.8 | 15.3 | 32.4 | n.d. | n.d. | n.d. | n.d. | 32.4 |
| CR112 | n.d. | 2.26 | 20.6 | 14.6 | 37.6 | n.d. | n.d. | n.d. | n.d. | 37.6 |
| CR114 | n.d. | 1.58 | 4.01 | 4.30 | 10.3 | n.d. | n.d. | n.d. | n.d. | 10.3 |
| CR116 | n.d. | <MDL | 18.1 | 4.75 | 22.9 | n.d. | n.d. | n.d. | n.d. | 22.9 |
| CR117 | n.d. | 1.98 | 26.0 | 28.3 | 56.3 | n.d. | n.d. | n.d. | n.d. | 56.3 |
| CR120 | n.d. | <MDL | 22.0 | 10.2 | 32.2 | n.d. | n.d. | n.d. | n.d. | 32.2 |
| CR121 | n.d. | 1.99 | 19.2 | 9.09 | 30.4 | n.d. | n.d. | n.d. | n.d. | 30.4 |
| CR122 | n.d. | <MDL | 17.7 | 6.60 | 24.3 | n.d. | n.d. | n.d. | n.d. | 24.3 |
| CR123 | n.d. | 2.05 | 22.7 | 16.2 | 41.0 | n.d. | n.d. | n.d. | n.d. | 41.0 |
| CR124 | n.d. | 3.84 | 28.4 | 40.2 | 72.5 | n.d. | n.d. | n.d. | n.d. | 72.5 |
| CR127 | n.d. | 2.45 | 12.1 | 2.43 | 17.2 | n.d. | n.d. | n.d. | n.d. | 17.2 |
| CR128 | n.d. | 3.23 | 25.8 | 13.0 | 42.2 | n.d. | n.d. | n.d. | n.d. | 42.2 |
| CR130 | n.d. | 4.08 | 37.3 | 15.1 | 56.6 | n.d. | n.d. | n.d. | n.d. | 56.6 |
| CR131 | n.d. | 1.61 | 28.0 | 12.8 | 42.5 | n.d. | n.d. | n.d. | n.d. | 42.5 |
| CR132 | n.d. | 3.49 | 24.8 | 9.92 | 38.3 | n.d. | n.d. | n.d. | n.d. | 38.3 |
| CR134 | n.d. | 2.67 | 53.4 | 10.1 | 66.2 | n.d. | n.d. | n.d. | n.d. | 66.2 |
| CR135 | n.d. | 3.65 | 40.3 | 8.25 | 52.2 | n.d. | n.d. | n.d. | n.d. | 52.2 |
| CR136 | n.d. | 2.86 | 32.8 | 12.6 | 48.4 | n.d. | n.d. | n.d. | n.d. | 48.4 |
| CR137 | n.d. | 3.14 | 32.7 | 11.8 | 47.8 | n.d. | n.d. | n.d. | n.d. | 47.8 |
| CR138 | n.d. | 3.41 | 32.1 | 18.6 | 54.2 | n.d. | n.d. | n.d. | n.d. | 54.2 |
| CR139 | n.d. | 2.70 | 27.0 | 17.2 | 47.0 | n.d. | n.d. | n.d. | n.d. | 47.0 |
| CR140 | n.d. | 3.09 | 32.0 | 17.5 | 52.7 | n.d. | n.d. | n.d. | n.d. | 52.7 |
| CR141 | n.d. | 2.71 | 33.0 | 33.3 | 69.1 | n.d. | n.d. | n.d. | n.d. | 69.1 |
| CR142 | n.d. | 3.59 | 28.8 | 22.3 | 54.8 | n.d. | n.d. | n.d. | n.d. | 54.8 |
| CR144 | n.d. | 2.78 | 26.4 | 17.6 | 46.9 | n.d. | n.d. | n.d. | n.d. | 46.9 |
| CR145 | n.d. | 2.48 | 22.5 | 13.2 | 38.3 | n.d. | n.d. | n.d. | n.d. | 38.3 |
| CR146 | n.d. | 3.29 | 21.9 | 16.5 | 41.8 | n.d. | n.d. | n.d. | n.d. | 41.8 |
| CR148 | n.d. | 2.54 | 22.9 | 15.9 | 41.4 | n.d. | n.d. | n.d. | n.d. | 41.4 |
| CR154 | n.d. | 2.86 | 24.2 | 14.2 | 41.4 | n.d. | n.d. | n.d. | n.d. | 41.4 |
| CR155 | n.d. | 2.48 | 22.3 | 9.58 | 34.5 | n.d. | n.d. | n.d. | n.d. | 34.5 |
| CR156 | n.d. | 2.41 | 16.1 | 10.1 | 28.8 | n.d. | n.d. | n.d. | n.d. | 28.8 |
| CR159 | n.d. | <MDL | 21.0 | 11.7 | 32.7 | n.d. | n.d. | n.d. | n.d. | 32.7 |
| CR162 | n.d. | 1.71 | 15.0 | 4.20 | 21.0 | n.d. | n.d. | n.d. | n.d. | 21.0 |
| CR164 | n.d. | 2.76 | 9.87 | 5.62 | 18.5 | n.d. | n.d. | n.d. | n.d. | 18.5 |
| CR166 | n.d. | 1.79 | 28.5 | 21.7 | 52.1 | n.d. | n.d. | n.d. | n.d. | 52.1 |
| CR167 | n.d. | 1.80 | 29.5 | 26.5 | 57.9 | n.d. | n.d. | n.d. | n.d. | 57.9 |
| CR168 | n.d. | 2.13 | 33.6 | 18.2 | 53.0 | n.d. | n.d. | n.d. | n.d. | 53.0 |
| CR171 | n.d. | 3.24 | 27.8 | 13.5 | 44.6 | n.d. | n.d. | n.d. | n.d. | 44.6 |
| CR172 | n.d. | 2.64 | 32.3 | 19.2 | 54.3 | n.d. | n.d. | n.d. | n.d. | 54.3 |
| CR173 | n.d. | 4.36 | 27.0 | 8.22 | 39.7 | n.d. | n.d. | n.d. | n.d. | 39.7 |
| CR174 | n.d. | 3.94 | 38.3 | 19.6 | 62.0 | n.d. | n.d. | n.d. | n.d. | 62.0 |
| CR175 | n.d. | 3.05 | 26.8 | 16.0 | 46.0 | n.d. | n.d. | n.d. | n.d. | 46.0 |
| CR177 | n.d. | 4.37 | 13.2 | 7.43 | 25.3 | n.d. | n.d. | n.d. | n.d. | 25.3 |
| CR179 | n.d. | 3.03 | 7.56 | 4.94 | 15.9 | n.d. | n.d. | n.d. | n.d. | 15.9 |
| CR182 | n.d. | 2.70 | 27.2 | 16.0 | 46.0 | n.d. | n.d. | n.d. | n.d. | 46.0 |
| CR183 | n.d. | 4.28 | 37.6 | 21.3 | 63.4 | n.d. | n.d. | n.d. | n.d. | 63.4 |
| CR184 | n.d. | 4.54 | 25.4 | 13.9 | 44.1 | n.d. | n.d. | n.d. | n.d. | 44.1 |
| CR186_1 | n.d. | 2.67 | 23.6 | 11.0 | 37.3 | n.d. | n.d. | n.d. | n.d. | 37.3 |
| CR186_2 | n.d. | 2.37 | 13.9 | 9.97 | 26.4 | n.d. | n.d. | n.d. | n.d. | 26.4 |
| CR187 | n.d. | 3.45 | 28.9 | 19.6 | 52.1 | n.d. | n.d. | n.d. | n.d. | 52.1 |
| CR188 | n.d. | 2.60 | 35.6 | 32.5 | 70.8 | n.d. | n.d. | n.d. | n.d. | 70.8 |
| CR190 | n.d. | 11.9 | 34.0 | 22.0 | 68.3 | n.d. | n.d. | n.d. | n.d. | 68.3 |
| CR192 | n.d. | 4.05 | 21.3 | 10.4 | 35.9 | n.d. | n.d. | n.d. | n.d. | 35.9 |
| CR194 | n.d. | 4.53 | 31.5 | 27.6 | 63.7 | n.d. | n.d. | n.d. | n.d. | 63.7 |
| CR195 | n.d. | 2.08 | 7.80 | 8.18 | 18.3 | n.d. | n.d. | n.d. | n.d. | 18.3 |
| CR163 | n.d. | 2.42 | 15.0 | 19.1 | 36.7 | n.d. | n.d. | n.d. | n.d. | 36.7 |
| CR197 | n.d. | 3.52 | 25.2 | 11.3 | 40.2 | n.d. | n.d. | n.d. | n.d. | 40.2 |
| CR198 | n.d. | 3.16 | 24.4 | 13.8 | 41.4 | n.d. | n.d. | n.d. | n.d. | 41.4 |
| CR199 | n.d. | 6.15 | 73.9 | 12.7 | 92.8 | n.d. | n.d. | n.d. | n.d. | 92.8 |
| CR200 | n.d. | 3.13 | 24.4 | 14.4 | 42.1 | n.d. | n.d. | n.d. | n.d. | 42.1 |
| CR201 | n.d. | 4.49 | 27.3 | 10.9 | 42.8 | n.d. | n.d. | n.d. | n.d. | 42.8 |
| CR202 | n.d. | 4.81 | 27.5 | 12.3 | 44.8 | n.d. | n.d. | n.d. | n.d. | 44.8 |
| CR203 | n.d. | 5.03 | 32.3 | 17.6 | 55.1 | n.d. | n.d. | n.d. | n.d. | 55.1 |
| CR204 | n.d. | 3.93 | 7.34 | 6.90 | 18.7 | n.d. | n.d. | n.d. | n.d. | 18.7 |
| CR205 | n.d. | 2.51 | 4.64 | 6.48 | 14.2 | n.d. | n.d. | n.d. | n.d. | 14.2 |
| CR206 | n.d. | 8.54 | 70.1 | 27.2 | 106 | n.d. | n.d. | n.d. | n.d. | 106 |
| CR208 | n.d. | 6.53 | 65.1 | 13.7 | 85.4 | n.d. | n.d. | n.d. | n.d. | 85.4 |
| CR209 | n.d. | 11.5 | 127 | 76.2 | 215 | n.d. | n.d. | n.d. | n.d. | 215 |
| CR210 | n.d. | 8.37 | 86.5 | 100 | 195 | n.d. | n.d. | n.d. | n.d. | 195 |
| CR211 | n.d. | 2.78 | 4.67 | 7.77 | 15.8 | n.d. | n.d. | n.d. | n.d. | 15.8 |
| CR212 | n.d. | 3.05 | 4.69 | 5.61 | 14.0 | n.d. | n.d. | n.d. | n.d. | 14.0 |
| CR213 | n.d. | 1.86 | 2.47 | 4.12 | 9.21 | n.d. | n.d. | n.d. | n.d. | 9.21 |
| CR214 | n.d. | <MDL | 1.86 | <MDL | 1.86 | n.d. | n.d. | n.d. | n.d. | 1.86 |
| CR215 | n.d. | 25.1 | 12.3 | 0.40 | 39.8 | n.d. | n.d. | n.d. | n.d. | 39.8 |
| CR216 | n.d. | 4.32 | 4.18 | 4.68 | 14.2 | n.d. | n.d. | n.d. | n.d. | 14.2 |
| CR217 | n.d. | <MDL | 1.60 | n.d. | 1.60 | n.d. | n.d. | n.d. | n.d. | 1.60 |
| P1 | 6.84 | 36.4 | 55.4 | 55.7 | 154 | 0.34 | <MDL | n.d. | 0.34 | 155 |
| P2 | <MDL | 17.0 | 72.3 | 42.8 | 132 | 0.18 | <MDL | n.d. | 0.18 | 132 |
| C1 | 7.34 | 142 | 78.8 | 41.3 | 269 | 0.78 | 3.51 | 5.43 | 9.72 | 279 |
| C2 | 216 | 106 | 848 | 3,912 | 5,082 | 0.33 | n.d. | 6.30 | 6.63 | 5,089 |
| C3 | 16.4 | 10.4 | 28.9 | 54.6 | 110 | 1.11 | n.d. | n.d. | 1.11 | 111 |
| C4 | 6.04 | 25.1 | 23.6 | 35.0 | 89.7 | 0.47 | n.d. | n.d. | 0.47 | 90.2 |
| F1 | n.d. | 20.0 | 228 | 52.6 | 301 | n.d. | n.d. | n.d. | n.d. | 301 |

**SI9 SI8 – Microcontaminants in crumb rubber by country: comparison with other studies**

***SI8.1 – Total concentration of organic (PAHs, plasticizers and additives) and inorganic (metals) microcontaminants in CR in the 53 samples in common with Armada et al. (2022) and Graça et al. (2022)*** [CHI – Chile; GER – Germany; ITA – Italy; FRA – France; CRO – Croatia; POL – Poland; SWE – Sweden; ALB – Albania; FIN – Finland; GRE – Greece; SPA – Spain; POR – Portugal].

**Figure S5 –** Total concentration (ng.g^-1^) of 18 PAHs – adapted from Armada et al. (2022).

**Figure S6 –** Total concentration (ng.g^-1^) of 19 plasticizers – adapted from Armada et al. (2022).

**Figure S7 –** Total concentration (ng.g^-1^) of 5 additives – adapted from Armada et al. (2022).

**Figure S8 –** Total concentration (ng.g^-1^) of 30 metals and metalloids – adapted from Graça et al. (2022).

**SI9 – Crumb rubber samples from Estonia**

**Figure S9 –** Mean total concentration (ng.g^-1^) of VMSs in crumb rubber from indoor and outdoor fields of Estonia.

**Figure S10 –** Total concentration (ng.g^-1^) of VMSs in the outdoor crumb rubber samples from Estonia, distributed by granulometry.

**Figure S11 –** Total concentration (ng.g^-1^) of VMSs in the outdoor crumb rubber samples from Estonia, distributed by field age.

**SI10 – Statistical analysis: crumb rubber**

***SI10.1 – Origin of the crumb rubber samples***

**Table S8 –** Spearman correlation coefficients (*ρ*) and respective *p*-values for the different classes of microcontaminants in crumb rubber – considering all countries.

| **Spearman's Correlations** | | | | | | | | | |
| --- | --- | --- | --- | --- | --- | --- | --- | --- | --- |
| **Variable 1** | |  | | **Variable 2** | | **Spearman's *ρ*** | | ***p*-value** | |
| PAHs |  |  |  | VMSs |  | 0.381 | ** | 0.006 |  |
| Plasticizers |  |  |  | VMSs |  | -0.312 | * | 0.026 |  |
| Additives |  |  |  | VMSs |  | 0.479 | *** | <0.001 |  |
| Metals |  |  |  | VMSs |  | -0.233 |  | 0.099 |  |
|  | | | | | | | | | |
| **p* < 0.05, ** *p* < 0.01, *** *p* < 0.001 | | | | | | | | | |

**Table S9 –** Dunn post hoc comparisons for the country distribution (crumb rubber from synthetic turf fields).

| **Dunn's Post Hoc Comparisons - Country** | | | | | | | | | | | | | |
| --- | --- | --- | --- | --- | --- | --- | --- | --- | --- | --- | --- | --- | --- |
| **Comparison** | | **z** | | **W_i_** | | **W_j_** | | ***p*-value** | | **p_bonf_** | | **p_holm_** | |
| Chile - Croatia |  | 0.842 |  | 41.000 |  | 18.500 |  | 0.400 |  | 1.000 |  | 1.000 |  |
| Chile - Finland |  | 0.372 |  | 41.000 |  | 35.111 |  | 0.710 |  | 1.000 |  | 1.000 |  |
| Chile - France |  | 0.307 |  | 41.000 |  | 35.200 |  | 0.759 |  | 1.000 |  | 1.000 |  |
| Chile - Greece |  | 1.095 |  | 41.000 |  | 24.100 |  | 0.273 |  | 1.000 |  | 1.000 |  |
| Chile - Italy |  | 0.110 |  | 41.000 |  | 38.750 |  | 0.912 |  | 1.000 |  | 1.000 |  |
| Chile - Poland |  | -0.250 |  | 41.000 |  | 46.667 |  | 0.803 |  | 1.000 |  | 1.000 |  |
| Chile - Portugal |  | -2.422 |  | 41.000 |  | 70.615 |  | 0.015 | * | 1.000 |  | 0.926 |  |
| Chile - Serbia |  | -2.498 |  | 41.000 |  | 85.500 |  | 0.012 | * | 0.974 |  | 0.762 |  |
| Chile - Slovakia |  | 0.823 |  | 41.000 |  | 19.000 |  | 0.410 |  | 1.000 |  | 1.000 |  |
| Chile - Spain |  | -2.631 |  | 41.000 |  | 76.150 |  | 0.009 | ** | 0.664 |  | 0.545 |  |
| Chile - Sweden |  | -1.725 |  | 41.000 |  | 73.600 |  | 0.084 |  | 1.000 |  | 1.000 |  |
| Croatia - Finland |  | -0.616 |  | 18.500 |  | 35.111 |  | 0.538 |  | 1.000 |  | 1.000 |  |
| Croatia - France |  | -0.579 |  | 18.500 |  | 35.200 |  | 0.563 |  | 1.000 |  | 1.000 |  |
| Croatia - Greece |  | -0.210 |  | 18.500 |  | 24.100 |  | 0.834 |  | 1.000 |  | 1.000 |  |
| Croatia - Italy |  | -0.678 |  | 18.500 |  | 38.750 |  | 0.498 |  | 1.000 |  | 1.000 |  |
| Croatia - Poland |  | -0.894 |  | 18.500 |  | 46.667 |  | 0.371 |  | 1.000 |  | 1.000 |  |
| Croatia - Portugal |  | -2.084 |  | 18.500 |  | 70.615 |  | 0.037 | * | 1.000 |  | 1.000 |  |
| Croatia - Serbia |  | -2.379 |  | 18.500 |  | 85.500 |  | 0.017 | * | 1.000 |  | 1.000 |  |
| Croatia - Slovakia |  | -0.014 |  | 18.500 |  | 19.000 |  | 0.988 |  | 1.000 |  | 1.000 |  |
| Croatia - Spain |  | -2.253 |  | 18.500 |  | 76.150 |  | 0.024 | * | 1.000 |  | 1.000 |  |
| Croatia - Sweden |  | -1.909 |  | 18.500 |  | 73.600 |  | 0.056 |  | 1.000 |  | 1.000 |  |
| Finland - France |  | -0.005 |  | 35.111 |  | 35.200 |  | 0.996 |  | 1.000 |  | 1.000 |  |
| Finland - Greece |  | 0.695 |  | 35.111 |  | 24.100 |  | 0.487 |  | 1.000 |  | 1.000 |  |
| Finland - Italy |  | -0.176 |  | 35.111 |  | 38.750 |  | 0.861 |  | 1.000 |  | 1.000 |  |
| Finland - Poland |  | -0.502 |  | 35.111 |  | 46.667 |  | 0.615 |  | 1.000 |  | 1.000 |  |
| Finland - Portugal |  | -2.783 |  | 35.111 |  | 70.615 |  | 0.005 | ** | 0.420 |  | 0.361 |  |
| Finland - Serbia |  | -2.771 |  | 35.111 |  | 85.500 |  | 0.006 | ** | 0.435 |  | 0.368 |  |
| Finland - Slovakia |  | 0.597 |  | 35.111 |  | 19.000 |  | 0.550 |  | 1.000 |  | 1.000 |  |
| Finland - Spain |  | -2.964 |  | 35.111 |  | 76.150 |  | 0.003 | ** | 0.237 |  | 0.207 |  |
| Finland - Sweden |  | -2.000 |  | 35.111 |  | 73.600 |  | 0.045 | * | 1.000 |  | 1.000 |  |
| France - Greece |  | 0.587 |  | 35.200 |  | 24.100 |  | 0.557 |  | 1.000 |  | 1.000 |  |
| France - Italy |  | -0.153 |  | 35.200 |  | 38.750 |  | 0.878 |  | 1.000 |  | 1.000 |  |
| France - Poland |  | -0.455 |  | 35.200 |  | 46.667 |  | 0.649 |  | 1.000 |  | 1.000 |  |
| France - Portugal |  | -2.161 |  | 35.200 |  | 70.615 |  | 0.031 | * | 1.000 |  | 1.000 |  |
| France - Serbia |  | -2.408 |  | 35.200 |  | 85.500 |  | 0.016 | * | 1.000 |  | 0.946 |  |
| France - Slovakia |  | 0.561 |  | 35.200 |  | 19.000 |  | 0.575 |  | 1.000 |  | 1.000 |  |
| France - Spain |  | -2.374 |  | 35.200 |  | 76.150 |  | 0.018 | * | 1.000 |  | 1.000 |  |
| France - Sweden |  | -1.760 |  | 35.200 |  | 73.600 |  | 0.078 |  | 1.000 |  | 1.000 |  |
| Greece - Italy |  | -0.718 |  | 24.100 |  | 38.750 |  | 0.473 |  | 1.000 |  | 1.000 |  |
| Greece - Poland |  | -0.994 |  | 24.100 |  | 46.667 |  | 0.320 |  | 1.000 |  | 1.000 |  |
| Greece - Portugal |  | -3.804 |  | 24.100 |  | 70.615 |  | <0.001 | *** | 0.011 | * | 0.011 | * |
| Greece - Serbia |  | -3.447 |  | 24.100 |  | 85.500 |  | <0.001 | *** | 0.044 | * | 0.041 | * |
| Greece - Slovakia |  | 0.191 |  | 24.100 |  | 19.000 |  | 0.849 |  | 1.000 |  | 1.000 |  |
| Greece - Spain |  | -3.896 |  | 24.100 |  | 76.150 |  | <0.001 | *** | 0.008 | ** | 0.007 | ** |
| Greece - Sweden |  | -2.620 |  | 24.100 |  | 73.600 |  | 0.009 | ** | 0.686 |  | 0.554 |  |
| Italy - Poland |  | -0.300 |  | 38.750 |  | 46.667 |  | 0.764 |  | 1.000 |  | 1.000 |  |
| Italy - Portugal |  | -1.759 |  | 38.750 |  | 70.615 |  | 0.079 |  | 1.000 |  | 1.000 |  |
| Italy - Serbia |  | -2.099 |  | 38.750 |  | 85.500 |  | 0.036 | * | 1.000 |  | 1.000 |  |
| Italy - Slovakia |  | 0.661 |  | 38.750 |  | 19.000 |  | 0.509 |  | 1.000 |  | 1.000 |  |
| Italy - Spain |  | -1.979 |  | 38.750 |  | 76.150 |  | 0.048 | * | 1.000 |  | 1.000 |  |
| Italy - Sweden |  | -1.506 |  | 38.750 |  | 73.600 |  | 0.132 |  | 1.000 |  | 1.000 |  |
| Poland - Portugal |  | -1.159 |  | 46.667 |  | 70.615 |  | 0.247 |  | 1.000 |  | 1.000 |  |
| Poland - Serbia |  | -1.592 |  | 46.667 |  | 85.500 |  | 0.111 |  | 1.000 |  | 1.000 |  |
| Poland - Slovakia |  | 0.879 |  | 46.667 |  | 19.000 |  | 0.380 |  | 1.000 |  | 1.000 |  |
| Poland - Spain |  | -1.380 |  | 46.667 |  | 76.150 |  | 0.167 |  | 1.000 |  | 1.000 |  |
| Poland - Sweden |  | -1.069 |  | 46.667 |  | 73.600 |  | 0.285 |  | 1.000 |  | 1.000 |  |
| Portugal - Serbia |  | -0.984 |  | 70.615 |  | 85.500 |  | 0.325 |  | 1.000 |  | 1.000 |  |
| Portugal - Slovakia |  | 2.064 |  | 70.615 |  | 19.000 |  | 0.039 | * | 1.000 |  | 1.000 |  |
| Portugal - Spain |  | -0.583 |  | 70.615 |  | 76.150 |  | 0.560 |  | 1.000 |  | 1.000 |  |
| Portugal - Sweden |  | -0.182 |  | 70.615 |  | 73.600 |  | 0.855 |  | 1.000 |  | 1.000 |  |
| Serbia - Slovakia |  | 2.361 |  | 85.500 |  | 19.000 |  | 0.018 | * | 1.000 |  | 1.000 |  |
| Serbia - Spain |  | 0.582 |  | 85.500 |  | 76.150 |  | 0.560 |  | 1.000 |  | 1.000 |  |
| Serbia - Sweden |  | 0.570 |  | 85.500 |  | 73.600 |  | 0.569 |  | 1.000 |  | 1.000 |  |
| Slovakia - Spain |  | -2.234 |  | 19.000 |  | 76.150 |  | 0.025 | * | 1.000 |  | 1.000 |  |
| Slovakia - Sweden |  | -1.892 |  | 19.000 |  | 73.600 |  | 0.059 |  | 1.000 |  | 1.000 |  |
| Spain - Sweden |  | 0.148 |  | 76.150 |  | 73.600 |  | 0.882 |  | 1.000 |  | 1.000 |  |
|  | | | | | | | | | | | | | |
| * *p* < 0.05, ** *p* < 0.01, *** *p* < 0.001 | | | | | | | | | | | | | |

**Table S10 –** *p*-values from the Spearman correlation applied to the different classes of microcontaminants in crumb rubber – for each country.

|  | **Chile** | **Finland** | **France** | **Greece** | **Italy** | **Poland** | **Portugal** | **Spain** | **Sweden** |
| --- | --- | --- | --- | --- | --- | --- | --- | --- | --- |
| PAHs vs. VMSs | 0.594 | 0.006** | 0.783 | 1.000 | 0.683 | 1.000 | 1.000 | 1.000 | 0.683 |
| Plasticizers vs. VMSs | 0.974 | 0.700 | 0.950 | 1.000 | 0.133 | 1.000 | 0.333 | 1.000 | 0.450 |
| Additives vs. VMSs | 0.379 | 0.024* | 0.783 | 1.000 | 0.683 | 1.000 | 1.000 | 1.000 | 0.517 |
| Metals vs. VMSs | 0.766 | 0.707 | 0.783 | 0.333 | 0.017* | 0.333 | 1.000 | 1.000 | 0.950 |

* *p* < 0.05, ** *p* < 0.01, *** *p* < 0.001

***SI10.2 – Granulometry of the crumb rubber samples***

**Table S11 –** Dunn post hoc comparisons for the granulometry distribution (crumb rubber from synthetic turf fields).

| **Dunn's Post Hoc Comparisons - Class** | | | | | | | | | | | | | |
| --- | --- | --- | --- | --- | --- | --- | --- | --- | --- | --- | --- | --- | --- |
| **Comparison** | | **z** | | **W_i_** | | **W_j_** | | ***p*-value** | | ***p*_bonf_** | | ***p*_holm_** | |
| 1-2 mm - 2-3 mm |  | -2.764 |  | 53.092 |  | 75.042 |  | 0.006 | ** | 0.034 | * | 0.034 | * |
| 1-2 mm - <1 mm |  | -0.469 |  | 53.092 |  | 61.250 |  | 0.639 |  | 1.000 |  | 1.000 |  |
| 1-2 mm - >3 mm |  | -0.996 |  | 53.092 |  | 63.231 |  | 0.319 |  | 1.000 |  | 1.000 |  |
| 2-3 mm - <1 mm |  | 0.753 |  | 75.042 |  | 61.250 |  | 0.452 |  | 1.000 |  | 1.000 |  |
| 2-3 mm - >3 mm |  | 1.011 |  | 75.042 |  | 63.231 |  | 0.312 |  | 1.000 |  | 1.000 |  |
| <1 mm - >3 mm |  | -0.102 |  | 61.250 |  | 63.231 |  | 0.919 |  | 1.000 |  | 1.000 |  |
|  | | | | | | | | | | | | | |
| * *p* < 0.05, ** *p* < 0.01 | | | | | | | | | | | | | |

***SI10.3 – Pitch age***

**Table S12 –** Dunn post hoc comparisons for the pitch age distribution (crumb rubber from synthetic turf fields).

| **Dunn's Post Hoc Comparisons - Class** | | | | | | | | | | | | | |
| --- | --- | --- | --- | --- | --- | --- | --- | --- | --- | --- | --- | --- | --- |
| **Comparison** | | **z** | | **W_i_** | | **W_j_** | | ***p*-value** | | ***p*_bonf_** | | ***p*_holm_** | |
| 1-5 years - 5-10 years |  | 1.672 |  | 74.783 |  | 58.821 |  | 0.094 |  | 0.945 |  | 0.472 |  |
| 1-5 years - < 1 year |  | -0.783 |  | 74.783 |  | 85.222 |  | 0.434 |  | 1.000 |  | 1.000 |  |
| 1-5 years - > 10 years |  | 2.232 |  | 74.783 |  | 53.115 |  | 0.026 | * | 0.256 |  | 0.179 |  |
| 1-5 years - Unknown |  | 3.215 |  | 74.783 |  | 44.774 |  | 0.001 | ** | 0.013 | * | 0.013 | * |
| 5-10 years - < 1 year |  | -2.031 |  | 58.821 |  | 85.222 |  | 0.042 | * | 0.422 |  | 0.253 |  |
| 5-10 years - > 10 years |  | 0.618 |  | 58.821 |  | 53.115 |  | 0.537 |  | 1.000 |  | 1.000 |  |
| 5-10 years - Unknown |  | 1.588 |  | 58.821 |  | 44.774 |  | 0.112 |  | 1.000 |  | 0.472 |  |
| < 1 year - > 10 years |  | 2.448 |  | 85.222 |  | 53.115 |  | 0.014 | * | 0.144 |  | 0.115 |  |
| < 1 year - Unknown |  | 3.149 |  | 85.222 |  | 44.774 |  | 0.002 | ** | 0.016 | * | 0.015 | * |
| > 10 years - Unknown |  | 0.925 |  | 53.115 |  | 44.774 |  | 0.355 |  | 1.000 |  | 1.000 |  |
|  | | | | | | | | | | | | | |
| * *p* < 0.05, ** *p* < 0.01 | | | | | | | | | | | | | |

**SI11 – Concentration of VMSs in other materials**

**Table S13 –** Concentration of VMSs in other artificial turf infill materials – 7 coloured crumb rubber, 4 cork and 1 rubber/coconut fibre (ng.g^-1^).

| **Sample ID** | **Material** | **D3** | **D4** | **D5** | **D6** | **∑D3-D6** | **L3** | **L4** | **L5** | **∑L3-L5** | **Total** |
| --- | --- | --- | --- | --- | --- | --- | --- | --- | --- | --- | --- |
| CL1 | Coloured CR | n.d. | 2.54 | 28.8 | 32.0 | 63.4 | n.d. | n.d. | n.d. | n.d. | 63.4 |
| CL2 | Coloured CR | n.d. | 3.73 | 30.1 | 31.8 | 65.6 | n.d. | n.d. | n.d. | n.d. | 65.6 |
| CL3 | Coloured CR | n.d. | 2.78 | 41.6 | 39.6 | 83.9 | n.d. | n.d. | n.d. | n.d. | 83.9 |
| CL4 | Coloured CR | n.d. | 3.59 | 44.7 | 50.9 | 99.2 | n.d. | n.d. | n.d. | n.d. | 99.2 |
| CL5 | Coloured CR | n.d. | 2.29 | 20.2 | 26.9 | 49.4 | n.d. | n.d. | n.d. | n.d. | 49.4 |
| CL6 | Coloured CR | n.d. | 1.70 | 8.89 | 4.75 | 15.3 | n.d. | n.d. | n.d. | n.d. | 15.3 |
| CL7 | Coloured CR | n.d. | <MDL | 8.88 | 1.95 | 10.8 | n.d. | n.d. | n.d. | n.d. | 10.8 |
| CK1 | Cork | n.d. | 6.28 | 25.7 | 41.6 | 73.5 | n.d. | n.d. | n.d. | n.d. | 73.5 |
| CK2 | Cork | n.d. | <MDL | 5.74 | 2.06 | 7.79 | n.d. | n.d. | n.d. | n.d. | 7.79 |
| CK3 | Cork | n.d. | 2.26 | 14.2 | 1.17 | 17.7 | n.d. | n.d. | n.d. | n.d. | 17.7 |
| CK4 | Cork | n.d. | <MDL | 7.47 | 2.10 | 9.57 | n.d. | n.d. | n.d. | n.d. | 9.57 |
| RC1 | Rubber/coconut fibre | n.d. | 4.61 | 34.9 | 34.2 | 73.7 | n.d. | n.d. | n.d. | n.d. | 73.7 |

**Figure S12 –** Mean total concentration (ng.g^-1^) of VMSs in crumb rubber (CR) and alternative synthetic turf infill materials, distributed by type of material.

**SI12 – Statistical analysis: crumb rubber and alternative infill materials**

**Table S14 –** Dunn post hoc comparisons for the type of infill material distribution.

| **Dunn's Post Hoc Comparisons - Material** | | | | | | | | | | | | | |
| --- | --- | --- | --- | --- | --- | --- | --- | --- | --- | --- | --- | --- | --- |
| **Comparison** | | **z** | | **W_i_** | | **W_j_** | | ***p*-value** | | ***p*_bonf_** | | ***p*_holm_** | |
| Coloured CR - Commercial CR |  | -2.130 |  | 93.286 |  | 141.43 |  | 0.033 | * | 0.199 |  | 0.133 |  |
| Coloured CR - Cork |  | 1.925 |  | 93.286 |  | 42.250 |  | 0.054 |  | 0.325 |  | 0.163 |  |
| Coloured CR - Field CR |  | 1.438 |  | 93.286 |  | 69.680 |  | 0.150 |  | 0.903 |  | 0.301 |  |
| Commercial CR - Cork |  | 3.742 |  | 141.43 |  | 42.250 |  | <0.001 | *** | 0.001 | ** | <0.001 | *** |
| Commercial CR - Field CR |  | 4.371 |  | 141.43 |  | 69.680 |  | <0.001 | *** | <0.001 | *** | <0.001 | *** |
| Cork - Field CR |  | -1.277 |  | 42.250 |  | 69.680 |  | 0.201 |  | 1.000 |  | 0.301 |  |
|  | | | | | | | | | | | | | |
| * *p* < 0.05, ** *p* < 0.01, *** *p* < 0.001 | | | | | | | | | | | | | |

**SI13 – Human exposure parameters**

**Table S15 –** Parameters used to estimate oral VMS exposure from crumb rubber.

| **Parameter** | | **Value** | **Unit** |
| --- | --- | --- | --- |
| Relative oral bioaccessibility factor (B) | D4 | 0.280 | Unitless |
|  | D5 | 0.172 |  |
|  | D6 | 0.150 |  |
| Ingestion rate (IR) | | 50.0 | mg.day^-1^ |
| Exposure frequency (EF) | Young player | 16.5 | day.year^-1^ |
|  | Adult player | 24.0 |  |
|  | Worker | 16.0 |  |
| Body weight (BW) | Young player | 31.8 | kg |
|  | Adult player | 80.0 |  |
|  | Worker | 80.0 |  |

**Table S16 –** Parameters used to estimate dermal VMS exposure from crumb rubber.

| **Parameter** | | **Value** | **Unit** |
| --- | --- | --- | --- |
| Relative dermal bioaccessibility factor (ABS) | D4 | 5.00 × 10^-3^ | Unitless |
|  | D5 | 1.00 × 10^-2^ |  |
|  | D6 | 3.00 × 10^-5^ |  |
| Adherence factor (AF) | | 4.00 × 10^-2^ | mg.cm^-2^ |
| Exposed surface area (SA) | Young player | 3,824 | cm^2^ |
|  | Adult player | 7,325 |  |
|  | Worker | 7,325 |  |
